# Single‐Cell Transcriptome Atlas Reveals the Underlying Mechanism of Kynurenic Acid in the Regulation of Tumor Immune Microenvironment in Glioblastoma

**DOI:** 10.1002/advs.202507705

**Published:** 2025-11-12

**Authors:** Di Chen, Liming Sun, Jiajun Chen, Zi Ye, Xuqiang Zhu, Hongjiang Li, Lixin Wu, Guohua Zhao, Qinghao Zhang, Guangyi Jiang, Yuchen Ji, Yake Xue, Hongwei Li, Ruokun Chen, Hongwei Zheng, Rong Zeng, Dongming Yan, Yong Zhang, Xueyuan Li, Jing Yan

**Affiliations:** ^1^ Department of Neurosurgery The First Affiliated Hospital of Zhengzhou University Zhengzhou Henan 450052 China; ^2^ Shanghai Clinical Research and Trial Center Shanghai Tech University Shanghai 201210 China; ^3^ Department of Human Resources The First Affiliated Hospital of Zhengzhou University Zhengzhou Henan 450052 China; ^4^ Department of Scientific Research The First Affiliated Hospital of Zhengzhou University Zhengzhou Henan 450052 China; ^5^ Department of MRI The First Affiliated Hospital of Zhengzhou University Zhengzhou Henan 450052 China

**Keywords:** CyTOF, glioblastoma, kynurenic acid, scRNA‐seq, T cells

## Abstract

Tryptophan metabolism plays a critical role in glioblastoma (GBM), however, the regulatory functions of kynurenic acid (KYNA) in this context remain poorly understood. Using an in‐house clinical cohort, targeted metabolomic analysis revealed significantly downregulated KYNA levels in GBM tissues compared to non‐tumor brain tissues. Further investigation demonstrated that KYNA administration markedly reduced tumor burden in an orthotopic GBM mouse model. Through integrated cytometry by time‐of‐fight (CyTOF), single‐cell RNA sequencing (scRNA‐seq), proteome, and flow cytometry analyses, this study delineated the alterative tumor immune landscape following KYNA treatment. Specifically, KYNA remodeled the immunosuppressive myeloid compartment within the GBM microenvironment. Additionally, KYNA reversed T cell exhaustion signatures, enhanced cytotoxic function, thereby augmented anti‐tumor T cell responses. Notably, the anti‐tumor effects of KYNA are abrogated in T cell‐deficient mouse models (nude and Rag2‐/‐), confirming its dependence on adaptive immunity. In summary, this study highlights the therapeutic potential of KYNA in GBM and provides a comprehensive, multi‐omics‐based understanding of its immunomodulatory mechanisms.

## Graphical abstract

KYNA regulates the tumor immune microenvironment in glioblastoma. KYNA reprograms the balance between T cell exhaustion and activation to suppress glioblastoma progression through inhibiting exhaustion‐associated markers (such as PD‐1, LAG3, and TOX) and enhancing immune activation and cytotoxicity markers including granzymes GZMA and GZMB.

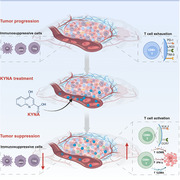

## 1 Introduction

Glioblastoma (GBM), a primary malignant neoplasm originating from glial cells within the cranial cavity, exhibits aggressive progression characterized by uncontrolled proliferation, diffuse infiltration, neovascularization, and the development of an immunosuppressive tumor microenvironment (TME).^[^ ^1^, ^2^, ^3^ ^]^ The highly invasive nature of GBM presents substantial therapeutic challenges in complete surgical removal or effective treatment, frequently leading to tumor recurrence and dismal clinical outcomes.^[^ ^4^, ^5^ ^]^ Despite incremental gains achieved through postoperative radiotherapy and chemotherapy, therapeutic efficacy remains constrained by the restrictive nature of the blood‐brain barrier (BBB) and blood‐tumor barrier (BTB), which impede adequate drug delivery to intracranial tumor sites.^[^ ^6^, ^7^ ^]^ Consequently, GBM patients typically face limited survival, high mortality rates, and unfavorable prognoses,^[^ ^8^ ^]^ underscoring the urgent need for innovative therapeutic strategies.

Tryptophan, an essential amino acid, is integral to several metabolic and physiological processes.^[^ ^9^, ^10^ ^]^ Tryptophan catabolism proceeds via three primary pathways: the kynurenine (KYN) pathway (KP), the serotonin (5‐hydroxytryptamine, 5‐HT) pathway, and the indole pathway.^[^ ^11^, ^12^ ^]^ Dysregulated tryptophan metabolism is frequently associated with oncogenic processes in GBM and other malignancies, often correlating with poor prognosis and aggressive tumor phenotypes.^[^ ^13^, ^14^ ^]^ Aberrations in tryptophan metabolic flux, particularly through the KP, contribute to tumor progression and immune evasion by fostering an immunosuppressive TME.^[^ ^15^, ^16^ ^]^ The KP generates a range of bioactive metabolites, including quinolinic acid, kynurenic acid (KYNA), anthranilic acid, and xanthurenic acid.^[^ ^17^ ^]^ Notably, activation of the KP leads to the production of immunosuppressive metabolites, such as kynurenine, which primarily modulate immune responses and promote tumorigenesis through interactions with the aryl hydrocarbon receptor.^[^ ^18^ ^]^

KYNA has long been recognized as a neuromodulator due to its inhibitory effects on endogenous ionotropic glutamate receptors and the *α*7‐nicotinic acetylcholine receptor (AHR). However, its role in GBM remains poorly understood. This study employed multi‐omics approaches to investigate the function of KYNA within the GBM tumor immune microenvironment. Using established orthotopic GBM mouse models, single‐cell RNA sequencing (scRNA‐seq), cytometry by time‐of‐flight (CyTOF), and proteomics were integrated to uncover the immunomodulatory mechanisms of KYNA and assess its therapeutic potential. Our findings offer mechanistic insights into KYNA‐mediated immune regulation and suggest a novel KYNA‐targeted therapeutic strategy for GBM treatment.

## 2 Results

### 2.1 KYNA Levels were Downregulated in GBM Tissues and KYNA Administration Mediated Anti‐Tumor Immunomodulatory Effects in an Orthotopic GBM Mouse Model

**Figure 1:**
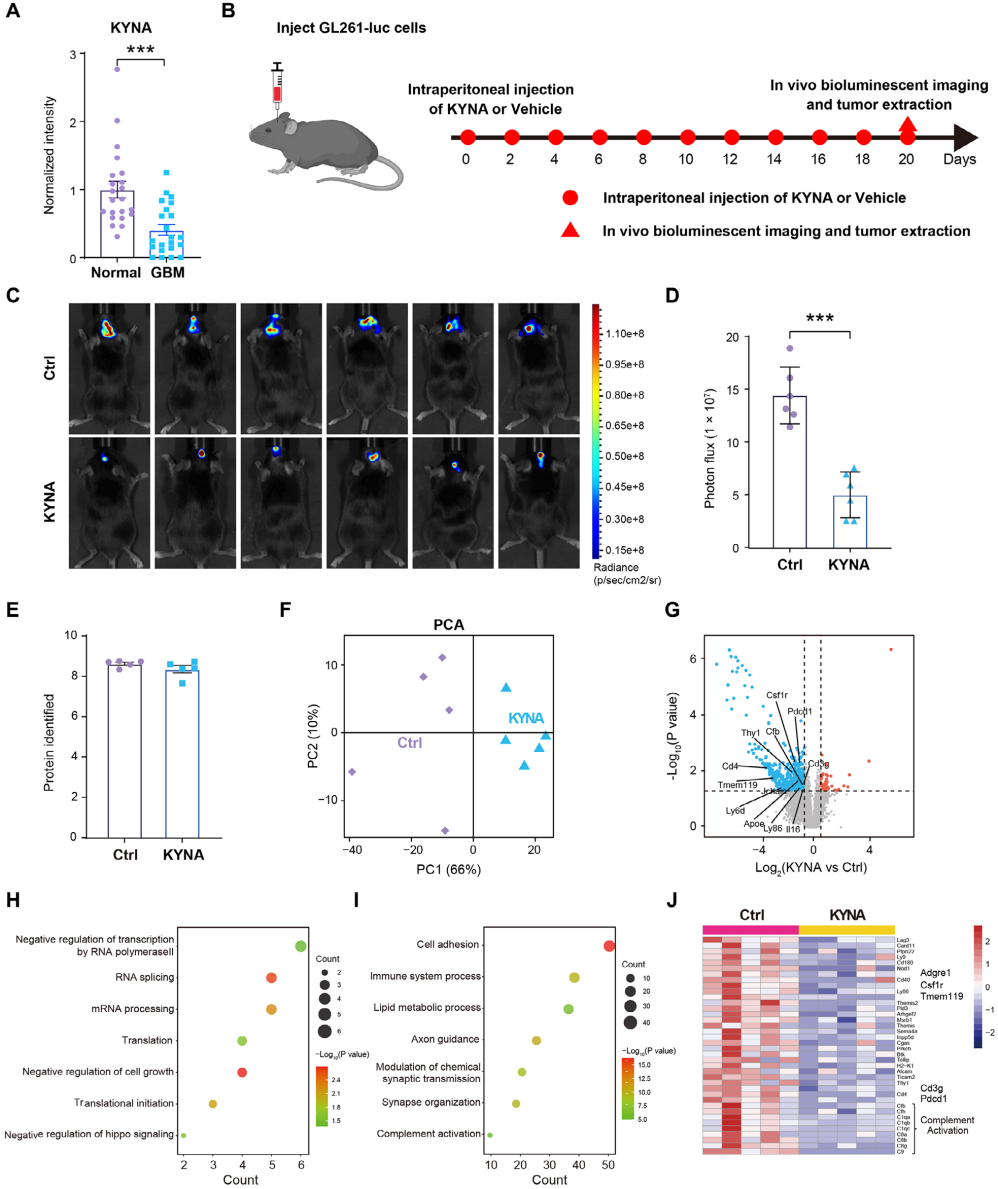
Multi‐omics analysis revealed the downregulation of KYNA in GBM and its immunomodulatory role in suppressing GBM progression. A) Targeted metabolomics analysis of KYNA levels in GBM and normal brain tissues (Normal = 22, GBM = 22). B) Experimental scheme for establishing an orthotopic GBM model using GL261‐luc cells and KYNA treatment. C) Bioluminescent imaging of tumor‐bearing mice in Ctrl or KYNA group. Ctrl, Vehicle‐treated mice; KYNA, KYNA‐treated mice (n = 6). D) Quantification of photon flux from bioluminescent imaging shown in (C) (n = 6). E) Number of proteins identified in GBM tissues in Ctrl or KYNA group (n = 5). F) Principal component analysis (PCA)‐of proteomic data from (E) (n = 5). G) Volcano plot of differentially expressed proteins (DEPs, p < 0.05, and |fold change| > 1.5) between KYNA and control groups. Representative immune‐related proteins are highlighted. H‐I) Gene Ontology (GO) analysis of biological processes for upregulated (H) and downregulated (I) proteins in the KYNA treatment group. J) Heatmap of DEPs enriched in immune system processes and complement activation (n = 5).

To elucidate the role of tryptophan and its downstream metabolites in GBM, a targeted metabolomic analysis was conducted on 22 GBM and 22 nontumor brain tissues obtained from a clinical cohort. The analysis revealed elevated levels of L‐tryptophan, L‐Kynurenine, Xanthurenic acid, and Nicotinamide in tumor tissues (Figure S1A–D, Supporting Information), whereas KYNA was markedly reduced in GBM samples (**Figure** **1**A). At the mRNA level, the enzymes involved in KYNA biosynthesis, including indoleamine 2,3‐dioxygenase 1 (IDO1), tryptophan 2,3‐dioxygenase‐2 (TDO2), kynurenine 3‐monooxygenase (KMO) and kynureninase (KYNU), were significantly upregulated in GBM tissues (Figure S1E,F, Supporting Information). However, kynurenine aminotransferase 1 (KYAT1), the key enzyme responsible for KYNA biosynthesis, was markedly downregulated in GBM tissues compared to adjacent normal tissues (Figure S1E,F, Supporting Information). Consistent findings were observed in the GBM data from the Chinese Glioma Genome Atlas (CGGA)‐325‐GBM database (Figure S1G, Supporting Information). Collectively, these results indicate that maintaining stable KYNA levels may be beneficial for protecting against GBM progression.

To assess the therapeutic potential of KYNA in GBM, an orthotopic GBM mouse model was established using GL261‐luc cells. KYNA was administered via intraperitoneal injection on alternate days (Figure 1B). Liquid chromatography‐tandem mass spectrometry (LC‐MS/MS) quantification confirmed successful KYNA delivery to orthotopic GBM tumors, with a mean D5‐KYNA concentration of 932.2 ng g^−1^ (Figure S2, Supporting Information), which is 84‐fold above the endogenous human GBM level (Figure S2, Supporting Information).^[^ ^19^ ^]^ By day 20, bioluminescence imaging indicated a significant reduction in tumor‐associated luminescent signals in KYNA‐treated mice compared to controls (Figure 1C,D), supporting the anti‐tumor efficacy of KYNA in GBM.

Next, the diagnostic and prognostic potential of KYNA was evaluated in clinical cohorts. In cohort 1, the diagnostic value of KYNA was 0.83, indicating a high diagnostic potential (Figure S3A, Supporting Information). In cohort 2, patients with higher KYNA levels exhibited longer survival times, suggesting that increasing KYNA content in GBM could inhibit the progression of GBM (Figure S3B, Supporting Information). Meanwhile, higher KYAT1 expression in GBM was associated with a better prognosis in both The Cancer Genome Atlas (TCGA) and CGGA‐325‐GBM database (Figure S3C,D, Supporting Information). These findings demonstrated that KYNA exhibited potent anti‐tumor efficacy and its level served as a significant prognostic predictor for GBM patients.

In order to investigate the mechanism of KYNA in GBM, we performed a 4D data independent acquisition (4D‐DIA) proteomics strategy on tumor tissues from control and KYNA‐treated groups. On average, 8880 proteins were quantified per sample (Figure 1E). Spearman correlation analysis confirmed high reproducibility across biological replicates (Figure S4A, Supporting Information). Principal component analysis (PCA) revealed clear separation between the KYNA and control groups along the first principal component (Figure 1F), indicating distinct proteomic signatures. Differential expression analysis identified a set of significantly downregulated proteins in the KYNA group (fold change > 1.5; p < 0.05), including immune‐related markers such as Cd3g, Pdcd1, and Jchain (Figure 1G). Gene Ontology (GO) enrichment analysis showed that upregulated proteins in the KYNA group were primarily involved in RNA splicing, translation, and negative regulation of cell growth (Figure 1H), while downregulated proteins were enriched in processes related to cell adhesion, immune system process, complement activation, and lipid metabolic process (Figure 1I). Notably, immune‐related proteins including myeloid cell markers (F4/80, Csf1r, Tmem119), T cell markers (Cd3g, Cd4), and exhaustion markers (Pdcd1, Lag3) were identified among the differentially expressed proteins (DEPs) (Figure 1J; Figure S4B,C, Supporting Information), indicating substantial remodeling of both innate and adaptive immune system in response to KYNA treatment. These results suggest that KYNA exerts a significant inhibitory effect on GBM progression, potentially through immunomodulation within the TME.

### 2.2 CyTOF Illustrated the Changes in the Tumor Immune Landscape Following KYNA Treatment

**Figure 2:**
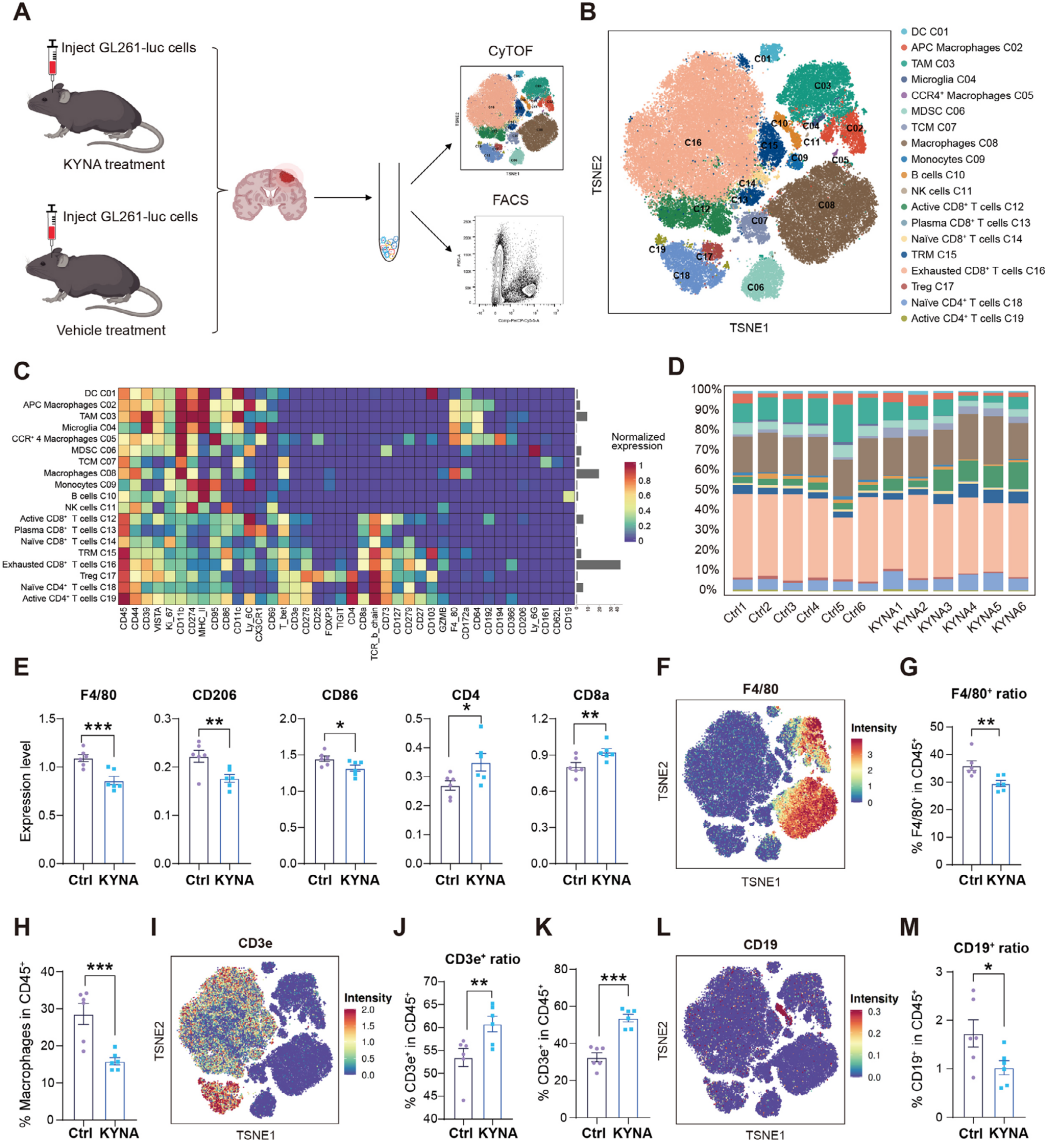
Comprehensive immune landscape remodeling in the GBM microenvironment following KYNA treatment. A) Experimental workflow for immune profiling in GBM mice by CyTOF and FACS (KYNA = 6, Ctrl = 6). B) t‐SNE analysis of defined cell clusters from CyTOF analysis of merged immune cells sorted. Populations are color‐coded. C) Average expression of immune markers in the identified cell populations. D) Stacked bar plot depicting the proportions of immune cell clusters, with colors matching those in (B) (n = 6). E) Bar plots showing the expression levels of myeloid markers and adaptive cell markers (n=6). F) t‐SNE plot displaying expression of the myeloid canonical marker F4/80. G,H) Quantification of F4/80^+^ cells in CD45^+^ cells by CyTOF (G) and FACS (H) (n = 6). I) t‐SNE plot illustrating the expression of the T cell marker CD3e. J,K) Quantification of CD3e^+^ cells in CD45^+^ cells by CyTOF (J) and FACS (K) (n = 6). L) t‐SNE plot showing the expression of the B cell marker CD19. M) Quantification of CD19^+^ cells in CD45^+^ cells by CyTOF (n = 6).

The tumor tissues collected from the KYNA and control groups of the murine GBM model were enzymatically dissociated into single‐cell suspensions and subsequently subjected to CyTOF and fluorescence‐activated cell sorting (FACS) analyses to delineate immune landscape alterations within the GBM TME induced by KYNA intervention (**Figure** **2**A; Figure S5A, Supporting Information). Following dimensionality reduction and unsupervised clustering, 19 distinct immune cell populations were identified via t‐distributed stochastic neighborhood embedding (t‐SNE) projection (Figure 2B), with cluster identities defined based on canonical biomarker expression (e.g., Treg, CD25^+^Foxp3^+^) (Figure 2C). Comparative analysis of immune subsets revealed substantial immunological remodeling in response to KYNA treatment (Figure 2D). Proteomic profiling had previously indicated differential expression of biomarkers associated with both myeloid cells and adaptive immune cells after KYNA treatment. These findings were corroborated using two independent analytical strategies in the CyTOF dataset. First, KYNA administration resulted in downregulation of myeloid‐associated markers (F4/80, CD206, CD86) and upregulation of adaptive immune markers (Cd4, Cd8a) (Figure 2E). Second, KYNA treatment markedly reduced the frequency of CD45^+^F4/80^+^ myeloid cells (Figure 2F,G), a trend confirmed by fluorescence‐based flow cytometry (Figure 2H). Within the adaptive compartment, KYNA increased the proportion of CD45^+^CD3e^+^ T cells while decreasing CD45^+^CD19^+^ B cells, with flow cytometric validation further substantiating T cell‐related observations (Figure 2I–M).

### 2.3 KYNA Remodeled the Immunosuppressive Myeloid Landscape and Augmented Anti‐Tumor T Cell Responses in GBM

**Figure 3:**
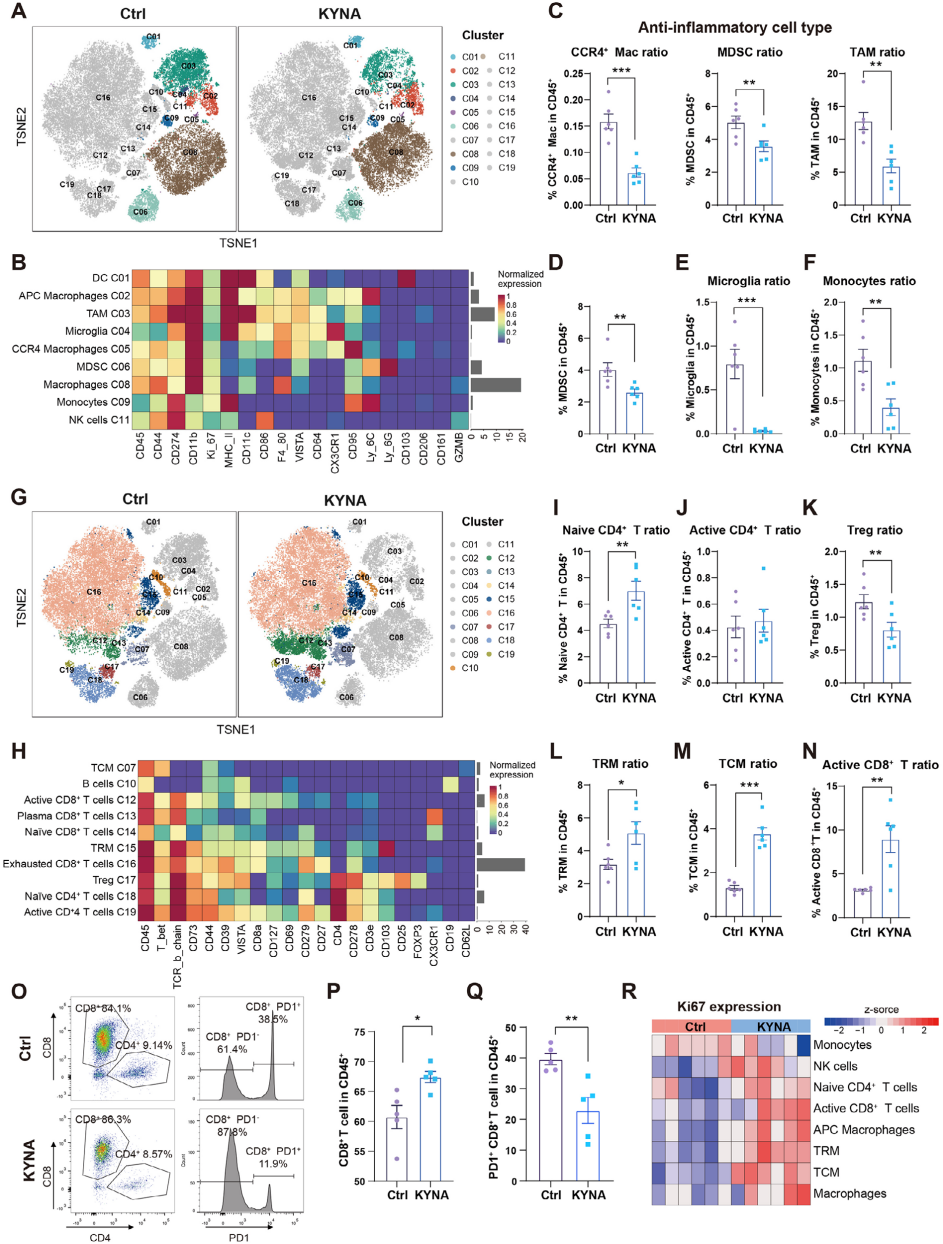
KYNA alleviated the immunosuppressive myeloid landscape and enhanced cytotoxic anti‐tumor T‐cell responses in GBM. A) t‐SNE plot visualizing the distribution of myeloid cell clusters among all immune cells. B) Heatmap illustrating marker expression across 9 myeloid cell clusters. C) Percentage change in anti‐inflammatory myeloid clusters within CD45^+^ cells (n = 6). D) Proportional change of MDSCs validated by FACS (n = 6). E,F) Percentage change in microglia (E) and monocyte (F) proportions within CD45^+^ cells (n = 6). G) t‐SNE plot displaying the distribution of T cell clusters within all immune cells. H) Heatmap showing marker expression among 10 T cell clusters. I‐K) Percentage change in CD4^+^ T cell proportions within CD45^+^ cells: naive CD4^+^ T cells (I), activated CD4^+^ T cells (J), and Treg cells (K) (n = 6). L‐N) Proportional change of CD8^+^ T cells within CD45^+^ cells: TRM (L), TCM (M), and active CD8^+^ T cells (N) (n = 6). O) Cell‐gating strategy and analysis of PD‐1 expression in CD8^+^ T cells (n = 5). P,Q) Proportion of CD8^+^ T cells (P) and PD‐1^+^CD8^+^ T cells (Q) within CD45^+^ cells in (O) (n = 5). R) Heatmap illustrating cell populations with differential Ki67 expression (n = 6).

To further delineate the impact of KYNA on distinct immune cell subsets, CyTOF data were stratified into myeloid and adaptive immune compartments based on cell‐type annotations. Unsupervised clustering via t‐SNE was employed to visualize the distribution of myeloid populations among the broader immune landscape (**Figure** **3**A). This analysis identified nine myeloid subtypes according to characteristic marker expression: dendritic cells (DCs), antigen‐presenting cells (APC) macrophages, tumor‐associated macrophages (TAMs), microglia, CCR4^+^ macrophages, myeloid‐derived suppressor cells (MDSCs), macrophages, monocytes, and natural killer (NK) cells (Figure 3B). KYNA administration did not significantly alter the ratio of total macrophages or major APCs (including DCs, APC macrophages, and NK cells) (Figure S5B,C, Supporting Information). However, a marked reduction was observed in the proportion of anti‐inflammatory myeloid cell populations within GBM‐bearing mice following KYNA treatment (Figure 3C). Notably, CCR4^+^ macrophages, known to express immunosuppressive mediators and to synergize with other myeloid subsets in establishing a tolerogenic TME, were diminished. Prior studies have shown that MDSCs and TAMs cooperatively reinforce immunosuppression in GBM through distinct molecular pathways, thereby impeding anti‐tumor immunity and promoting tumor progression.^[^ ^20^ ^]^ This study confirmed a significant decrease in MDSC frequency in KYNA‐treated mice, as validated by FACS (Figure 3D). As the primary immune cells within the central nervous system, activated microglia are closely linked to neuroimmune dysregulation. Consistent with reductions in other anti‐inflammatory myeloid subsets, both microglia and monocytes were significantly diminished post‐KYNA intervention (Figure 3E,F). These cell types are known contributors to T cell exhaustion via secretion of inhibitory cytokines and expression of immune checkpoint ligands.^[^ ^21^ ^]^ Such immunosuppressive interactions are particularly pronounced within the TME, where persistent antigenic stimulation induces functional exhaustion of adaptive immunity. Collectively, these results suggest that KYNA mitigates the abundance of tumor‐promoting myeloid populations that drive immune evasion and T cell dysfunction, underscoring its role in remodeling the myeloid compartment of the TME.

The antitumor efficacy of T cells in GBM is frequently attenuated, predominantly due to exhaustion, functional impairment, and modulation by the immunosuppressive TME. To delineate the heterogeneity of adaptive immune subsets following KYNA treatment, cluster analysis was performed. A t‐SNE plot illustrated the distribution and relative percentages of adaptive immune cell clusters (Figure 3G), followed by classification into ten distinct clusters based on established canonical markers (Figure 3H). Notably, KYNA treatment resulted in a significant expansion of naive CD4^+^ T cells (Figure 3I), a subset essential for initiating adaptive responses and preserving immune equilibrium. In contrast, no statistically significant increase was observed in tumoricidal CD4^+^ effector T cells (Figure 3J). A pronounced decrease in regulatory T (Treg) cells, which contribute to immunosuppression and restrict CD8^+^ T cell infiltration in the TME, was also detected (Figure 3K). Statistical comparisons further revealed significant elevations in three functionally distinct T cell subsets in the KYNA‐treated group compared to control group: tissue‐resident memory T (TRM) cells associated with localized immune coordination (Figure 3L), central memory T (TCM) cells responsible for sustained immunological memory (Figure 3M), and cytotoxic CD8^+^ T cells with antitumor activity (Figure 3N). The proportion of plasma CD8^+^ T cells was significantly elevated in the KYNA‐treated group compared to controls (Figure S5D, Supporting Information). FACS analysis confirmed that KYNA administration substantially reshaped T cell composition relative to the control group (Figure 3O), with a pronounced increase in CD8^+^ T cell frequency (Figure 3P). Although CyTOF data indicated a non‐significant reduction in exhausted T cell proportions in the KYNA group (Figure S5E, Supporting Information), fluorescence‐based flow cytometry revealed a notable decline in PD1^+^CD8^+^ T cells (Figure 3Q). These results imply that KYNA may confer dual immunomodulatory effects by simultaneously expanding cytotoxic T cell subsets and diminishing exhaustion‐associated phenotypes. To assess immune cell proliferative dynamics within the TME of GBM, Ki‐67 expression was quantified across immune populations (Figure 3R). Elevated Ki‐67 levels were observed in T cells and macrophages with antitumor immunostimulatory functions, suggesting enhanced proliferative capacity in these subsets. In contrast, immunosuppressive monocytes exhibited significantly reduced Ki‐67 expression, indicating diminished proliferative potential.

Comprehensive CyTOF and FACS analyses of GBM tissue‐resident immune cells demonstrated that KYNA treatment induced a decline in pro‐tumorigenic and immunosuppressive populations, including TAMs, MDSCs, and Tregs, while enriching immunostimulatory subsets such as TRM, TCM, and activated CD8^+^ T cells. These shifts underscore the profound modulatory impact of KYNA on the immune landscape of GBM.

### 2.4 scRNA‐seq Analysis Revealed KYNA‐Driven Myeloid Subset Remodeling with Dual Anti‐Tumor Effects in GBM

**Figure 4:**
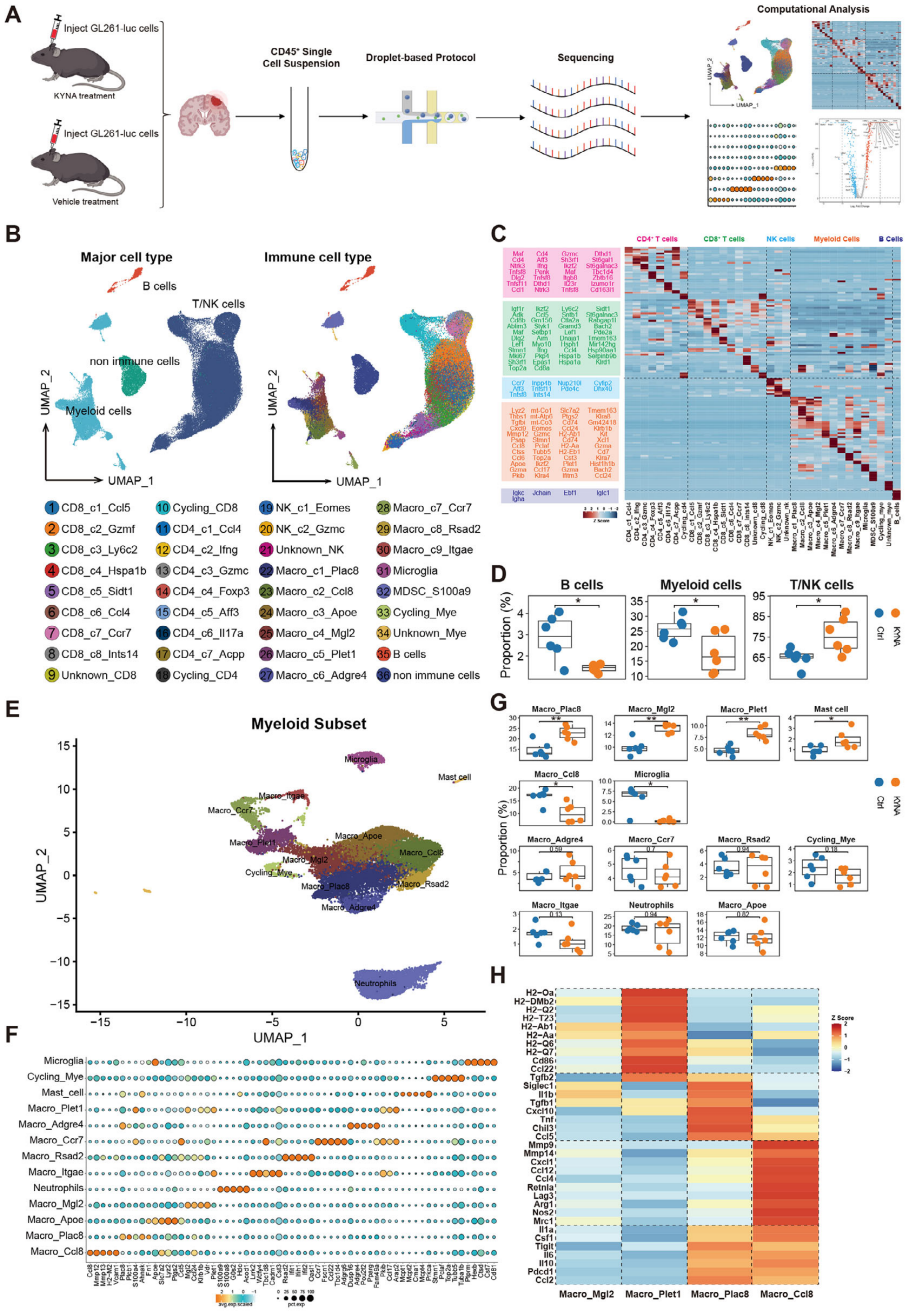
scRNA‐seq profiling revealed KYNA‐driven remodeling of myeloid and adaptive immune landscapes in GBM. A) Schematic overview of the experimental design and analysis workflow for scRNA‐seq (KYNA = 6, Ctrl = 6). B) Uniform manifold approximation and projection (UMAP) plot visualizing clusters of major cell types. C) Expression patterns of signature genes in distinct GBM‐infiltrating immune cell clusters. D) Quantification of B cell, myeloid cell, and T/NK cell proportions between KYNA and control groups (n = 6). E) UMAP plot showing the 13 identified myeloid clusters. F) Expression patterns of selected marker genes in myeloid clusters. G) Box plots showing the proportions of myeloid clusters (n = 6). H) Heatmap illustrating characteristic genes across four statistically different myeloid cell populations.

To validate the immunological alterations induced by KYNA identified through CyTOF and FACS analyses, and to further delineate its cell type‐specific effects, scRNA‐seq was performed using the 10 × Genomics platform on CD45^+^ immune cells isolated from KYNA‐treated and control groups (**Figure** **4**A). Following quality control filtering, a total of 85377 high‐quality immune cells were obtained, comprising T/NK cells (including CD4^+^ T cells, CD8^+^ T cells, and NK cells), myeloid cells, and B cells (Figure 4B,C). In alignment with CyTOF and FACS findings, KYNA treatment led to a significant reduction in B cell and myeloid cell populations, alongside a notable expansion of T/NK cell clusters (Figure 4D). Unsupervised clustering of the scRNA‐seq data identified 13 distinct myeloid subpopulations (Figure 4E,F). Comparative analysis revealed statistically significant shifts in four macrophage subsets—Macro_Plac8, Macro_Mgl2, Macro_Plet1, and Macro_Ccl8—characterized by high expression of Plac8, Mgl2, Plet1, and Ccl8, respectively. Post‐KYNA treatment, anti‐tumor mast cells were significantly increased. These cells are known to inhibit GBM cell proliferation and migration via GSK3β downregulation and STAT3 pathway suppression.^[^ ^22^ ^]^ Microglial populations exhibited a marked decline in the KYNA‐treated group, corroborating observations from CyTOF analysis (Figure 4G). Further statistical interrogation highlighted substantial remodeling of tumor‐associated myeloid compartments. The Macro_Plet1 subset upregulated major histocompatibility complex‐II (MHC‐II)‐related genes (*H2‐Oa, H2‐DMb2, Cd86*), indicative of enhanced antigen‐presenting functionality. Macro_Plac8 demonstrated elevated expression of pro‐inflammatory and tumoricidal genes (*Il1b, Tnf, Ccl5*). In contrast, Macro_Ccl8, which was downregulated following treatment, predominantly expressed immunosuppressive genes such as *Lag3, Arg1, and Nos2* (Figure 4H).

CD4^+^ T cell subset analysis via unsupervised clustering identified 8 distinct subsets (Figure S6A,B, Supporting Information). The proportions of the CD4_c1_Ccl4 and CD4_c2_Ifng subsets were significantly increased in the KYNA‐treated group (Figure S6C,D, Supporting Information). Ccl4, produced by CD4^+^ T cells, could recruit effector CD8^+^ T cells, thereby enhancing immunity.^[^ ^23^ ^]^ Secretion of interferon‐gamma (IFN‐*γ*) was a hallmark of cytotoxic CD4^+^ T cells and could recruit and activate other cytotoxic immune cells.^[^ ^24^, ^25^ ^]^ forkhead box P3 (Foxp3) was a marker of Tregs and possessed immunosuppressive functions; however, its proportion was significantly decreased in the KYNA‐treated group (Figure S6C,D, Supporting Information). This remodeling of CD4^+^ T cell heterogeneity toward enhanced effector functions and reduced suppression contributes to the overall immunostimulatory reprogramming of the GBM microenvironment by KYNA.

### 2.5 KYNA Treatment Correlated with Enhanced CD8^+^ T Cell Phenotypes Associated with Anti‐Tumor Function

**Figure 5:**
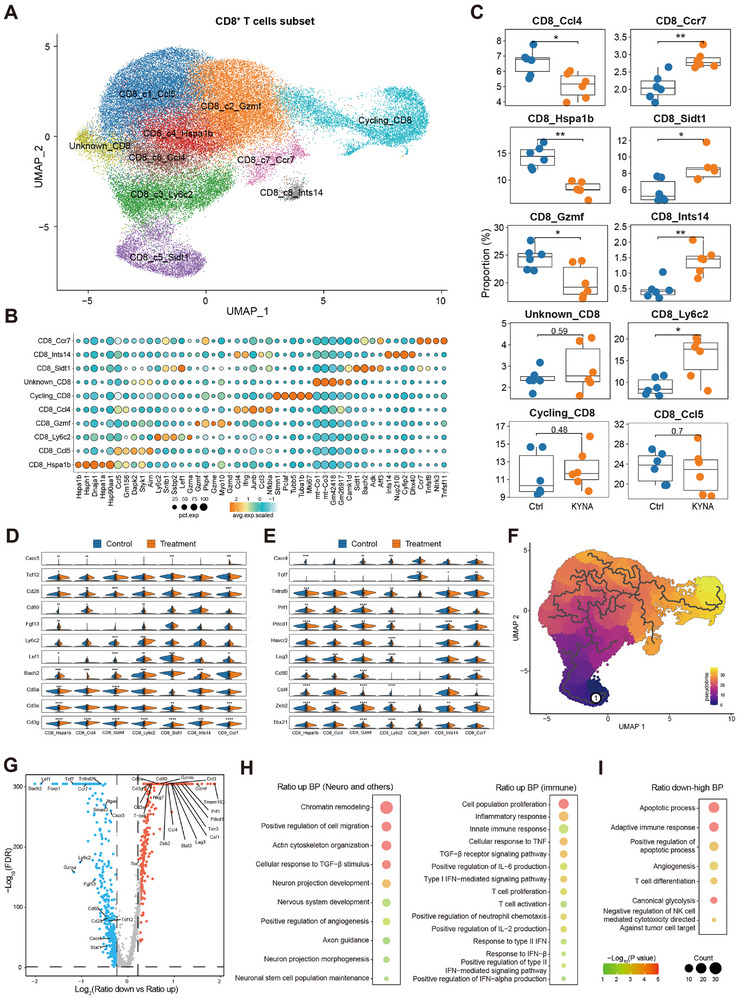
KYNA reprogrammed CD8^+^ T cell heterogeneity to alleviate exhaustion and amplify cytotoxic functions in GBM. A) UMAP visualization of CD8^+^ T cell clusters. B) Dot plot exhibiting the expression of characteristic marker genes in CD8^+^ T cell clusters. C) Box plots quantifying the proportions of CD8^+^ T cell clusters (n = 6). D,E) Violin plots displaying functional gene expression in CD8^+^ T cells. F) Pseudotime trajectory analysis of CD8^+^ T cell differentiation. G) Volcano plot showing differentially expressed genes (DEGs, p < 0.05, and |fold change| > 1.2) in KYNA‐regulated elevated versus decreased CD8^+^ T cells. H,I) GO analysis of biological processes for upregulated genes (H) and downregulated genes (I).

To elucidate KYNA‐mediated regulation of CD8^+^ T cell phenotypic heterogeneity, unsupervised clustering analysis of single‐cell transcriptomes was performed, identifying ten transcriptionally distinct CD8^+^ T cell subsets (**Figure** **5**A,B). Box plot analysis revealed significant KYNA‐induced modulation of exhaustion dynamics within the CD8^+^ T cell compartment (Figure 5C). Notably, exhaustion‐associated clusters—including Ccl4^+^CD8^+^ T cell subset, Hspa1b^+^CD8^+^ T cell subset, and Gzmf^+^CD8^+^ T cell subset—were significantly reduced in the KYNA‐treated group compared to controls. Conversely, memory‐precursor populations, such as Ccr7^+^ and Sidt^+^ CD8^+^ T cells, as well as Ly6c2^+^ effector‐like subsets, were markedly expanded (Figure 5C).^[^ ^26^, ^27^ ^]^ Previous studies have implicated Ccl4 and Gzmf in T cell dysfunction and terminal differentiation, while elevated expression of heat shock protein genes such as *Hspa1b* has been associated with exhaustion‐like phenotypes.^[^ ^28^, ^29^, ^30^ ^]^ Further analysis identified significant transcriptomic differences across six subsets exhibiting altered proportions in response to KYNA treatment (Figure 5D,E). Violin plots demonstrated that effector‐associated genes (e.g., *Ly6c2*) were significantly upregulated, whereas exhaustion‐related markers (*Pdcd1*, *Lag3*) were downregulated.

Pseudotime trajectory analysis suggested enrichment of Sidt^+^ subsets in early differentiation states following KYNA treatment, consistent with enhanced stem‐like properties (Figure 5F). A volcano plot highlighted differentially expressed genes (DEGs) between proportionally increased and decreased T cell clusters following KYNA treatment (Figure 5G). Expanded clusters exhibited elevated expression of stemness‐related genes (*Tcf7*, *Ccr7*), activation markers (*Ly6c2*, *Cd28*), and cytotoxic effectors (Gzma), whereas reduced clusters were enriched for exhaustion‐associated transcripts, including *Tim3*, *Lag3*, and *Pdcd1*. GO enrichment analysis identified distinct biological processes (BP) associated with upregulated and downregulated DEGs (Figure 5H,I). Upregulated DEGs were significantly enriched in neural development processes, including axon guidance and neuroprotection, as well as immune‐related pathways such as T cell proliferation, activation, inflammatory response, and tumor‐killing effects. In contrast, downregulated DEGs were primarily enriched in apoptosis‐related pathways and angiogenesis regulation. scRNA analysis demonstrated KYNA‐mediated regulation of CD8^+^ T cell heterogeneity. KYNA reduced exhaustion‐associated subsets (Ccl4^+^, Hspa1b^+^, Gzmf^+^) while expanding memory‐precursor (Ccr7^+^, Sidt^+^) and effector (Ly6c2^+^) populations. Upregulated genes included effector molecules (*Ly6c2*, *Cd28*, *Gzma*), while exhaustion markers (*Pdcd1*, *Lag3*) were downregulated. Pseudotemporal trajectory analysis revealed enhanced stem‐like properties. Differentially expressed genes were enriched in neural development and immune activation (upregulated genes), and apoptosis/angiogenesis (downregulated genes). Collectively, these data suggested that KYNA promoted CD8^+^ T cell states characterized by reduced exhaustion signatures, enhanced effector potential, and stem‐like properties, which might contribute to improved anti‐tumor function.

### 2.6 KYNA Reprograms the Immunosuppressive Tumor Microenvironment by Reversing T Cell Exhaustion and Enhancing Cytotoxic Function

Building upon our findings that KYNA reprograms tumor‐infiltrating lymphocytes in GBM, the specific immune cell type targeted by KYNA within the GBM microenvironment remains undefined, given its pleiotropic effects on multiple immune populations.^[^ ^31^, ^32^ ^]^ Our results demonstrated that KYNA exhibited no direct effect on GL261 cell proliferation in vitro, ruling out a tumor cell‐intrinsic mechanism (Figure S7A, Supporting Information). To investigate the role of adaptive immunity in KYNA's function, we established GL261‐based GBM models in immunodeficient mice lacking adaptive immune cells, including Balb/c‐Nude mice (T cell‐deficient) and Rag2‐/‐mice (T and B cell‐deficient). KYNA treatment significantly reduced the survival of tumor‐bearing Balb/c‐Nude mice (Figure S7B, Supporting Information), and promoted GBM progression in Rag2‐/‐mice compared to the Ctrl group (Figure S7C,D, Supporting Information). These findings indicated that the anti‐tumor efficacy of KYNA was dependent on the presence of adaptive immune cells.

**Figure 6:**
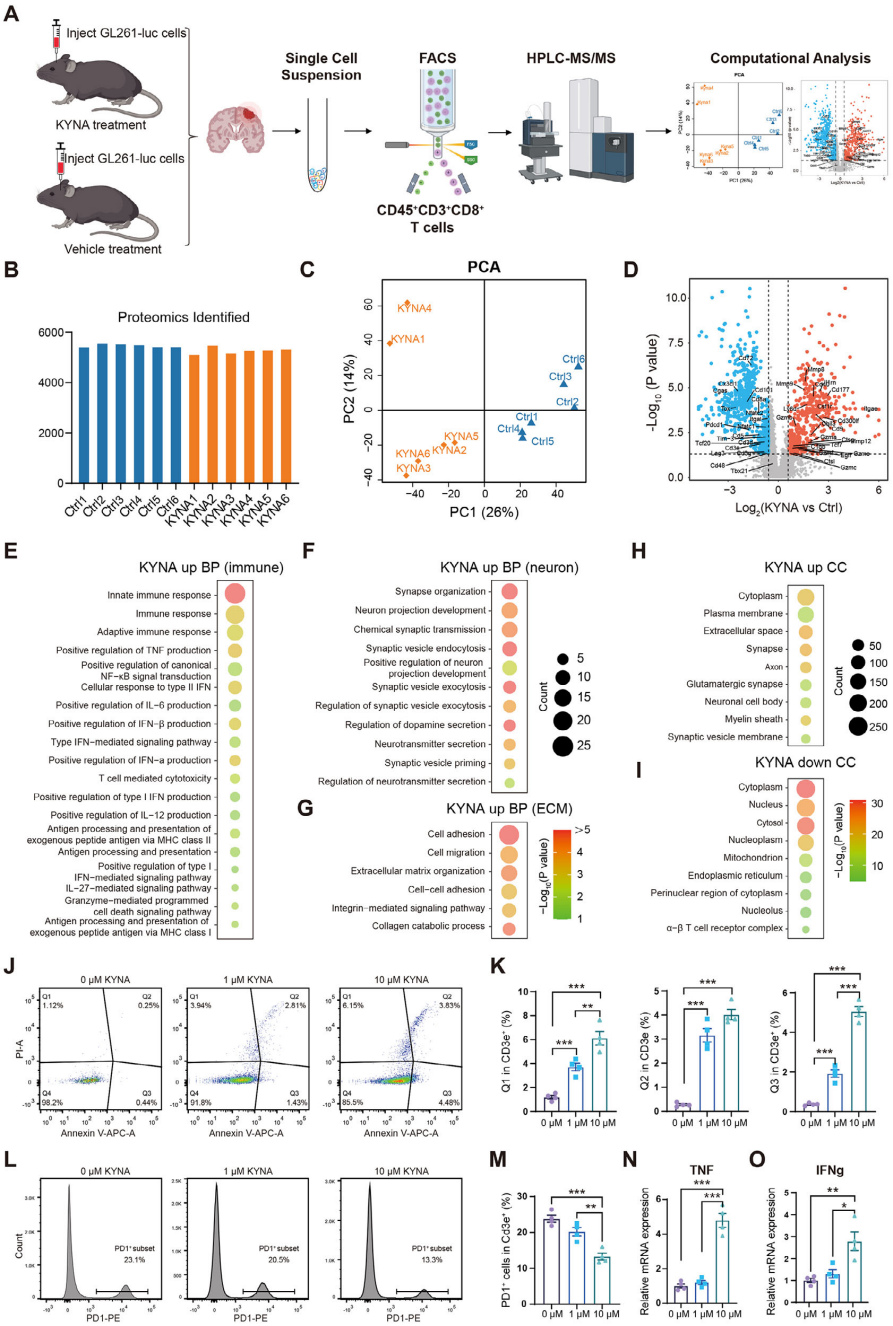
Integrated proteomics and in vitro experiments revealed the reversal of T cell exhaustion and enhanced cytotoxic function mediated by KYNA. A) Workflow for 4D‐DIA proteomics analysis of CD45^+^Cd3e^+^CD8^+^ T cells (KYNA = 6, Ctrl = 6). B) Number of proteins identified in CD8^+^ T cells (n = 6). C) PCA of proteomic profiles (n = 6). D) Volcano plot of differentially expressed proteins (DEPs, p < 0.05, and |fold change| > 1.5) between CD8^+^ T cells. Representative exhausted markers are highlighted. E‐I) GO enrichment analysis of KYNA‐regulated DEPs in immune‐related biological processes (E), neuro‐related biological processes (F), ECM‐related biological processes (G), and cellular components (H‐I). J) Flow cytometry analysis of apoptosis in exhausted CD8^+^ T cells treated with different concentrations of KYNA (n = 4). K) Flow cytometry quantification of the proportion of different apoptotic states of exhausted T cells in (J) (n = 4). L) Representative FACS gating showing the frequency of PD‐1^+^CD8^+^ T cells after treatment with different concentrations of KYNA (n = 4). M) Pooled data showing the frequency of PD‐1^+^CD8^+^ T cells treated with different concentrations of KYNA (n = 4). N,O) RT‐qPCR analysis of TNF and IFN‐*γ* in exhausted CD8^+^ T cells treated with different concentrations of KYNA (n = 4).

To further investigate the regulatory effects of KYNA on GBM‐resident T cells at the protein level, 4D‐DIA proteomic analysis was performed on CD45^+^Cd3e^+^CD8^+^ T cells sorted from GBM tissues of KYNA‐treated and control mice (**Figure** **6**A). Proteomic profiling identified over 5000 proteins per sample (Figure 6B). PCA demonstrated high biological reproducibility and clear separation between KYNA‐treated and control groups, indicating distinct proteomic signatures (Figure 6C). Comparative analysis of T cell proteomes revealed patterns consistent with the scRNA‐seq data. Exhaustion‐associated markers—such as PDCD1, TIM‐3, LAG3, Cgas, and TOX—were significantly downregulated, while immune activation and cytotoxicity markers—including granzymes Gzma, Gzmb, Gzmc, Gzme, and Gzmf—were markedly upregulated in the KYNA group (Figure 6D). Owing to the deeper detection sensitivity of proteomics compared to transcriptomics, additional differentially expressed proteins were identified. GO enrichment analysis of upregulated proteins in KYNA‐treated samples revealed three major functional categories: immune‐related, neuron‐related, and extracellular matrix (ECM)‐related pathways (Figure 6E–G). In the immune‐related cluster, upregulated proteins were enriched in tumor‐killing pathways, including positive regulation of TNF/IL‐6/IFN production, antigen processing and presentation, granzyme‐mediated apoptotic signaling, and T cell‐mediated cytotoxicity (Figure 6E). These results suggest that KYNA reprograms the balance between T cell exhaustion and activation in GBM, enhancing anti‐tumor effector functions. Furthermore, elevated TOX expression is closely associated with T cell exhaustion and critically dependent on nuclear factor of activated T cells (NFAT) family engagement.^[^ ^33^, ^34^ ^]^ Our data revealed concomitant reductions in Tox, Nfatc1, and Nfatc2 expression following KYNA treatment (Figure S8, Supporting Information).

The neuron‐related enrichment highlighted pathways involved in nerve repair and neurotransmission, such as synapse organization, neuron projection development, and chemical synaptic transmission (Figure 6F), implying a potential neuroprotective role for T cells in the KYNA‐treated group. ECM‐related enrichment indicated enhanced T cell infiltration following KYNA treatment (Figure 6G). Cellular component analysis of differentially expressed proteins further supported these findings. Upregulated proteins in the KYNA‐treated group were predominantly localized to the extracellular space, synapse, axon, and myelin sheath (Figure 6H), while upregulated proteins in the control group were enriched in the nucleus, mitochondria, and *α*–*β* T cell receptor complex (Figure 6I), reflecting a functional shift in T cells following KYNA exposure.

Integrated scRNA‐seq and proteomic analyses indicated that KYNA treatment was associated with increasing apoptotic signatures and enhancing cytotoxic protein expression in T cells. To test the effect of KYNA on apoptosis of exhausted T cells, we treated exhausted T cells with different concentrations of KYNA and detected the apoptosis. KYNA treatment correlated with increased apoptosis in exhausted T cells in vitro (Figure 6J,K), implying a potential role in selectively depleting this compartment. Concurrently, we observed reduced PD‐1^+^CD8^+^ T cells and elevated TNF/IFN‐*γ* expression in T cell exhaustion cell model (Figure 6L‐O).

## 3 Discussion

GBM is an aggressive brain tumor characterized by a highly immunosuppressive TME, which significantly impairs the effectiveness of therapeutic strategies.^[^ ^35^ ^]^ The metabolism of tryptophan plays a critical role in the TME of GBM, with the KP serving as the primary catabolic route for L‐tryptophan.^[^ ^13^, ^36^ ^]^ This pathway generates several important metabolites, including KYN, neuroprotective agents such as KYNA, excitotoxic quinolinic acid, and essential pyridinucleotides like nicotinamide adenine dinucleotide (NAD).^[^ ^37^, ^38^ ^]^ The initial step in the KP is catalyzed by IDO‐1, indoleamine 2,3‐dioxygenase‐2 (IDO‐2), and TDO‐2.^[^ ^39^ ^]^ The upregulation of IDO‐1 and TDO‐2 within the TME contributes significantly to the inhibition of anti‐tumor immune responses.^[^ ^40^, ^41^ ^]^ The conversion of tryptophan to KYNA through IDO and TDO‐mediated pathways is a key mechanism that establishes an immunosuppressive microenvironment, promoting systemic immune tolerance.^[^ ^42^, ^43^, ^44^ ^]^ Enhanced tryptophan metabolism not only depletes local tryptophan, limiting T‐cell proliferation and functionality, but also generates immunosuppressive metabolites that further hinder anti‐tumor immunity.^[^ ^45^, ^46^ ^]^

As a key terminal metabolite of the tryptophan‐kynurenine pathway, KYNA is primarily synthesized by KYAT1‐catalyzed conversion of KYN.^[^ ^47^, ^48^ ^]^ And it generally does not undergo further oxidation to downstream metabolites. Although this metabolic process occurs in various tissues, however, renal excretion is the dominant route of its in vivo clearance.^[^ ^31^ ^]^ Specifically, KYNA is highly bound to plasma proteins (≈99%), which impedes effective glomerular filtration, leading to a reliance on active tubular transport for elimination. In this process, organic anion transporters 1 and 3 (OAT1/OAT3) play a central role by mediating KYNA uptake from the blood into renal tubular epithelial cells, followed by transcellular transport into the lumen via multidrug resistance‐associated protein 4 (MRP4).^[^ ^49^ ^]^ Collectively, these mechanisms support that tumor‐detected signals derive from the unmetabolized D5‐KYNA prototype.

KYNA may also contribute to tumor progression by influencing neuroinflammation and affecting the infiltration and function of immune cells within the TME.^[^ ^50^, ^51^ ^]^ For example, in gastric cancer, KYNA induces ferroptosis in NK cells, leading to their exhaustion and further promoting an immunosuppressive TME.^[^ ^52^ ^]^ In non‐small cell lung cancer, cancer‐associated fibroblasts synthesize KYN, which activates the AHR, triggering AKT (protein kinase B, PKB) and extracellular signal‐regulated kinase (ERK) signaling pathways that enhance cell proliferation and resistance to epidermal growth factor receptor tyrosine kinase inhibitors.^[^ ^53^ ^]^ In GBM, elevated TDO‐2 expression drives KYN synthesis and AHR activation, which not only enhances cancer cell motility but also suppresses the proliferation and function of immune cells in the TME.^[^ ^15^ ^]^ Ongoing clinical trials are exploring novel therapies targeting IDO‐1, TDO‐2, and AHR across various cancers.^[^ ^54^ ^]^ Specifically, Phase I and Phase II trials are evaluating IDO‐1 inhibitors for GBM treatment.^[^ ^55^ ^]^

T cell exhaustion is a key mechanism by which tumors evade immune detection, characterized by a progressive decline in T cell function. This decline is marked by reduced effector activity, an increase in inhibitory receptors, and metabolic alterations.^[^ ^56^, ^57^, ^58^ ^]^ KYNA plays a critical role in the onset and progression of T cell exhaustion through various mechanisms.^[^ ^59^ ^]^ In oral squamous cell carcinoma (OSCC), KYNA influences cell populations within the TME by primarily promoting the expansion of neutrophils. This leads to increased interleukin‐1*β* (IL‐1*β*) production, which further expands neutrophils and induces CD8^+^ T cell exhaustion, ultimately facilitating OSCC progression.^[^ ^60^ ^]^ Additionally, KYNA impairs T cell antitumor responses by inhibiting the secretion of crucial cytokines, such as interleukin‐2 (IL‐2) and IFN‐*γ*.^[^ ^61^ ^]^

In this study, targeted metabolomics analysis revealed a significant reduction in KYNA expression in GBM tissues compared to normal counterparts. To further investigate the role of KYNA in GBM, this study developed mouse models of GBM and administered KYNA according to a defined experimental protocol. Subsequent luciferase imaging and tumor extraction demonstrated a substantial reduction in tumor size following KYNA treatment, resulting in an improved overall condition of the GBM models. Moreover, cytokine microarray analysis revealed significant changes in cytokine expression, including Csf1r and Pdcd1, following KYNA treatment. GO enrichment analysis indicated that the differentially expressed cytokines were primarily associated with pathways regulating the negative regulation of cell growth and complement activation.

Additionally, significant changes in the expression of immunologically relevant proteins were observed following KYNA administration, offering a more comprehensive view of its impact on the TME of GBM. Notably, the proportion of anti‐inflammatory myeloid cells, including CCR4^+^ macrophages, MDSCs, and TAMs, significantly decreased. CCR4^+^ macrophages are known to facilitate immune evasion by tumor cells, modulate angiogenesis, and promote invasion and metastasis.^[^ ^62^ ^]^ These macrophages also secrete chemokines that recruit immunosuppressive cells, such as Tregs and MDSCs, into the TME, further impairing anti‐tumor immune responses.^[^ ^63^, ^64^ ^]^ In the analysis of adaptive immune cell clustering, KYNA treatment resulted in a marked increase in naive T cells and a decrease in Tregs. Upon activation, naive T cells differentiate into effector T cells, which are responsible for tumor cell destruction, while Tregs suppress immune responses and aid in immune evasion.^[^ ^65^, ^66^ ^]^ The significant increase in CD8^+^ T cell populations post‐KYNA treatment, coupled with the downregulation of PD‐1 expression on CD8^+^ T cells, suggests that KYNA may enhance the cytotoxic T cell response while mitigating exhaustion markers. scRNA‐seq further revealed a reduction in tumor‐promoting pre‐myeloid cells and an increase in tumor‐killing myeloid cells following KYNA administration. Analysis of CD8^+^ T cells showed upregulation of effector‐related genes (e.g., *Ly6c2*) and downregulation of exhaustion markers (e.g., *Pdcd1* and *Lag3*). Additionally, T cell activation markers (*Ly6c2*, *Cd28*) and cytotoxic effectors were significantly increased.

Interestingly, we observed a completely contrasting effect of KYNA on tumor progression between Wild‐type (WT) and immunodeficient mice. KYNA administration significantly reduced the tumor burden in Wild‐type mice but promoted tumor progression in Balb/c‐Nude and Rag2^−/−^ models. In vitro assays confirmed KYNA alone exerted no direct pro‐ or anti‐tumor activity, indicating its pro‐tumor effect in Rag2^−/−^ mice was possibly associated with immunoregulation, particularly through modulating myeloid cells. Miyamoto et al. reported that gut microbiota‐derived KYNA facilitated the recruitment of GPR35^+^ macrophages.^[^ ^67^ ^]^ Furthermore, Pagano et al. demonstrated that GPR35 activation on macrophages promoted tumor growth by stimulating angiogenesis.^[^ ^68^ ^]^ However, our scRNA‐seq data of WT mice showed that KYNA treatment significantly reduced immunosuppressive myeloid cell subsets, which normally promote tumor progression and inhibit T cell activity. These findings suggested that the effect of KYNA on tumor progression was highly related to the tumor immune environment, especially the involvement of T cells. Therefore, the effect of KYNA on myeloid cells cannot be simply summarized as “inhibitory” or “activating”, but should be combined with the immune status in the tumor microenvironment.

This study has several limitations. First, despite achieving an intratumoral KYNA concentration that was 84‐fold higher than the endogenous baseline, the overall biodistribution efficiency to the GBM was low (≈ 0.23%). This underscores the challenge of drug delivery to intracranial tumors and suggests that the observed efficacy might be further enhanced with optimized delivery strategies. Second, our study focused on the intact D5‐KYNA molecule and did not investigate its potential downstream metabolites. Although KYNA is frequently considered a presumed terminal metabolite in the tryptophan pathway, evidence suggests it can be further metabolized into products like quinaldic acid. However, the biological relevance of quinaldic acid remains largely elusive.^[^ ^69^ ^]^ Thus, the potential contribution of these downstream metabolites to the observed effects cannot be excluded. Third, while multi‐omics analyses indicated an immunomodulatory role for KYNA on T cells, its precise molecular targets and underlying mechanisms on these cells remain incompletely defined. Finally, the regulatory effect of KYNA on myeloid cells in the absence of adaptive immunity has not been sufficiently explored. Future studies should profile the metabolic fate of KYNA in the tumor microenvironment and elucidate the systematic mechanisms responsible for its contrasting effects in WT and T cell‐deficient models.

In summary, this study provides a comprehensive understanding of KYNA's immunomodulatory role in GBM through multi‐omics analysis. KYNA reduces tumor burden and reshapes the TME by promoting cytotoxic T cell activity while reducing exhaustion markers. These findings underscore the therapeutic potential of KYNA in GBM treatment and offer new insights into its complex immune‐regulatory mechanisms. Further exploration of KYNA's molecular targets and its clinical application will advance the management of GBM.

## 4 Experimental Section

### GBM Samples Collection

This study conforms to the guidelines issued in the Declaration of Helsinki and was approved by the Ethics Committee of the First Affiliated Hospital of Zhengzhou University (FAHZZU, Approval Number: 2023‐KY‐1455‐001). Informed consent was obtained from all patients for the use of fresh tumor specimens. Patient samples were collected at FAHZZU between March 2023 and July 2025 and allocated to two cohorts: Cohort 1 comprised 22 GBM tissues (acquired from surgical resections) and 22 non‐tumor brain tissues (obtained during decompressive procedures in traumatic brain injury cases) for tryptophan metabolite quantification; Cohort 2 included 60 GBM patients to analyse the association between KYNA levels and clinical prognosis.

### Detection of Tryptophan Metabolites

UPLC‐MS/MS with MRM (multiple reaction monitoring) mode was performed to detect tryptophan and its metabolites in cohort 1.^[^ ^70^ ^]^ Briefly, tissue samples were homogenized in pre‐cooled 80% methanol, then vortexed and centrifuged (20000 ×g, 4 °C). The supernatant was lyophilized and reconstituted. Chromatography was performed using an Agilent Poroshell 120 EC‐C18 column at 40 °C, with 5 mmol L^−1^ ammonium acetate (with 0.01% formic acid) and acetonitrile as mobile phases. An AB Sciex 4500MD mass spectrometer was used for detection. Target metabolites were identified and quantified using standard compounds. The criteria for differential metabolites were defined by multivariate thresholds (variable importance in projection >1) and univariate criteria (p < 0.05, Fold Change > 2 or < 0.5).

For the detection of D5‐KYNA, a method based on previous studies was employed with minor modifications.^[^ ^19^ ^]^ It administered D5‐KYNA via intraperitoneal injection to GBM‐bearing mice (orthotopic GL261 model) and sham‐operated mice and detected D5‐KYNA levels specifically in GBM tumor tissues by targeted mass spectrometry on Q‐Exactive (Thermo Fisher Scientific). The separation of analytes was achieved using the C18 HPLC column (Thermo Fisher Scientific Hypersil GOLD VANQUISH C18, 100 mm X 2.1, size 1.9 µm) on a 5 min gradient elution. The Q1–Q3 ion transitions for D5‐KYNA were 195.2/149.0.

### Cell Line and Cell Culture

The GL261 cell line was obtained from the Chinese Academy of Sciences Cell Bank and the GL261‐luc cell line was obtained from the Cellverse company Limited. In accordance with standardized protocols, GL261 and GL261‐luc cell lines were cultured in Dulbecco's Modified Eagle's Medium (DMEM, Thermo Fisher Scientific, USA), which was supplemented with 10% fetal bovine serum (FBS, Thermo Fisher Scientific, USA) and 1% penicillin‐streptomycin (Thermo Fisher Scientific, USA). The continuous culture of these cells was performed in a sterile environment at a temperature of 37 °C with a 5% CO_2_ concentration.

### Animals and Mouse Model

Wild‐type C57BL/6, Rag2‐/‐, and Balb/c‐Nude male mice, aged 8 to 10 weeks, were procured from Liaoning Changsheng Biotechnology Co., Ltd. (Liaoning, China) and housed in the Experimental Animal Center of Zhengzhou University, where they were maintained under specific pathogen‐free conditions. The ambient temperature was maintained between 22 and 25 °C, and the mice were subjected to a 12‐h light/dark cycle, with unrestricted access to food and water. All animal experiments were performed in compliance with the Guidelines for the Care and Use of Experimental Animals established by the National Scientific Research Commission and received approval from the Animal Ethics Committee at Zhengzhou University (ZZU‐LAC20240906).

To create an orthotopic model of GBM, the animals were anesthetized using isoflurane, and an analgesic was injected before surgery. Before performing stereotaxic intracranial injection, the surgical site was prepared by shaving and disinfecting with 70% ethanol. Following this, an incision was made along the midline, and a 1‐mm diameter puncture was created in the right parietal region, positioned 2 mm posterior to the coronal suture and 2 mm lateral to the sagittal suture. The anesthetized mice were securely positioned on a stereotaxic frame, and 4 × 10^5^ GL261‐Luc cells were injected intracranially to a depth of 3 mm using a 26‐gauge needle, with the cells suspended in 2.5 microliters of normal saline. Upon completion of the injection, the needle was carefully withdrawn, and the skin was sutured using 4‐0 nylon thread.

Following the implantation surgery, a total of 12 tumor‐bearing mice were randomly assigned to two distinct groups: an experimental group and a control group, each comprising 6 mice. The experimental group received intraperitoneal injections of KYNA (Cat# S4719, Selleck, USA) at a dosage of 25 mg kg^−1^, whereas the control group received an equal volume of dimethyl sulfoxide (DMSO) (diluted 1:10 in saline). Injections were administered at 2‐day intervals. On day 20 after implantation, the mice were humanely euthanized and bioluminescent imaging was used to assess tumor growth. Fresh GBM tissue from mice was collected for subsequent analysis. All animal experiments were performed in accordance with randomized and blinded protocols to ensure the integrity of the results.

### In Vitro Induction of CD8^+^ T Cell Exhaustion

CD8^+^ T cells were isolated from the spleen in wild‐type OT‐I (C57BL/6 background) using negative selection with magnetic beads (Milteny, 130‐104‐075). To generate exhausted CD8^+^ T cells, purified OT‐I CD8^+^ T cells were co‐cultured with CD11b^+^CD11c^−^ monocytes (0.1%) and CD11b^+^CD11c^+^ dendritic cells (0.1%) in complete RPMI 1640 medium supplemented with 5 ng mL^−1^ IL‐15 (Thermo Fisher Scientific, 210‐15‐10UG) and 5 ng mL^−1^ IL‐7 (Thermo Fisher Scientific, 217‐17‐10UG), along with 10 ng mL^−1^ OVA257‐264 peptide (MCE, 138831‐86‐4) for 48 h. Following initial stimulation, cells were washed and subjected to daily restimulation with OVA257‐264 peptide in the presence of cytokines and KYNA (0, 1, or 10 µM) for an additional 72 h. On day 5, all cell populations were harvested and subsequently used for Annexin V‐APC/PI Apoptosis Detection assay (Vazyme, A214‐01), FACS detection and qPCR.

### Flow Cytometry

The brain single‐cell suspension and exhausted CD8^+^ T cells were processed as previously described. Cells were centrifuged at 500 × g for 3 min at 4 °C and resuspended in 100 µL of FACS buffer (PBS containing 0.5% BSA and 2 mM EDTA, pH 7.5). Cell suspensions were incubated with fluorophore‐conjugated antibodies for 25 min at 4 °C in the dark. Excess antibodies were removed by washing with FACS buffer. For cell sorting, cell viability was assessed by DAPI staining to exclude dead cells and were measured on a BD FACS Aria III (BD Biosciences) using application settings and at least 10000 cells were collected. For cell analysis, the cells were performed on a CytoFlex LX (Beckman). The data were analyzed using FlowJo software. The FACS anti‐mouse antibodies used were: Anti‐CD45‐APC (Biolegend, 147708), Anti‐CD45‐PerCP/Cyanine5.5 (Biolegend, 157208), Anti‐CD11b‐FITC (Biolegend, 101206), Anti‐F4/80‐PE (Biolegend, 111604), Anti‐Gr1‐BV605 (Biolegend, 108440), Anti‐CD11b‐APC/Cy7 (Biolegend, 101226), Anti‐CD4‐PE/Cy7 (Biolegend, 100422), Anti‐CD8a‐PE (Biolegend, 100708), Anti‐PD1‐APC (Biolegend, 1385210).

### Quantitative Real‐Time Polymerase Chain Reaction (RT‐qPCR)

Total RNA was extracted from GBM tissue and exhausted CD8^+^ T cells using Trizol reagent (Invitrogen). Complementary DNA (cDNA) was synthesized using the iScript cDNA synthesis kit (Bio‐Rad). qPCR was performed in 20 µL reactions containing SYBR Green Supermix (Bio‐Rad) on a CFX96 system (Bio‐Rad) under standard cycling conditions. Gene expression was normalized to GAPDH using the 2^(‐ΔΔCt) method.

### Sample Preparation for MS‐Based Proteomics

Tissue samples and CD8^+^ T cells sorted by FACS were essentially as described.^[^ ^71^ ^]^ Brain tissues were digested by FASP‐protocol with trypsin (protein: trypsin = 1: 50) at 37 °C with gentle shaking for 16 h,^[^ ^72^ ^]^ and desalted by a three Empore SPE Disks C18 (3 M Purification). For sorted cells, the samples were lyophilized in a vacuum freeze dryer and dissolved in 100 µL ATC buffer solution (100 mmol L^−1^ Ammonium Bicarbonate (NH_4_HCO_3_), 5 mmol L^−1^ Tris (2‐carboxyethyl) phosphine hydrochloride (TCEP), 20 mmol L^−1^ 2‐Chloroacetamide (CAA), 3 µL trypsin, pH 8). After incubation at 56 °C for 1 h, the samples were also desalted by C18 tips. 300 ng peptides were used for further mass spectrometry analysis.

### Mass Spectrometry Analysis and Database Searching

All proteomic samples were performed on a Timstof‐Pro mass spectrometer (Bruker) using a 30‐min gradient and DIA mode. The parameters were described as previously.^[^ ^71^ ^]^ DIA data was searched by Spectronaut (version 18, Biognosys Inc.) using directDIA+ with the library of Uniprot Mus musculus (10090) (reviewed, 17038 proteins, 2024) and default parameters. After normalizing by median and imputing missing values by random forest (ntree = 100), Student's t‐test and mean ratio were used to identify differential proteins between each other (p value < 0.05 & |fold change| > 1.5). GO analysis of significant difference proteins was enriched on DAVID.^[^ ^73^ ^]^ All proteomic data analyses were performed using R platform (version 4.1.2).

### CyTOF

The single‐cell suspension was prepared according to the protocol provided by the manufacturer.^[^ ^74^ ^]^ Purified antibodies were conjugated with the designated metal tags employing the MaxPAR Antibody Labelling Kit (Fluidigm, USA). Before application, the conjugated antibodies underwent titration to ascertain the optimal concentration. After washing once with 1xPBS, cells were stained with 100 µL of 250 nM cisplatin (Fluidigm, USA) for 5 min on ice to eliminate dead cells. Following this, the cells were incubated in an Fc receptor blocking solution and then stained with a cocktail of surface antibodies for 30 min on ice. Before intracellular staining, cells were fixed in 200 µL of intercalation solution (Maxpar Fix and Perm Buffer with 250 nM 191/193Ir, Fluidigm) for an entire night after being washed twice with FACS buffer (1× PBS+0.5% bovine serum albumin). Intracellular staining was conducted by applying intracellular antibodies cocktail for 30 min on ice, according to the instructions provided by the manufacturer. Subsequently, the cells were subjected to a washing procedure and then resuspended in deionised water, combined with 20% EQ beads (Fluidigm, USA). The data were acquired on a Helios mass cytometer (Fluidigm, USA), normalized, and analyzed through manual gating with FlowJo software (version 10.8).

### scRNA‐seq

A single‐cell suspension containing more than 90% of live cells was obtained, and the cell concentration was adjusted to 1000 cells µL^−1^. The prepared single‐cell suspension was combined with a mixture of gel beads carrying unique barcodes and enzymes. Utilizing hydrodynamic principles, this mixture was processed through a microfluidic device known as a “double cross,” resulting in the formation of Gel Bead‐In‐Emulsions (GEMs). Effective GEMs encapsulate gel beads containing pre‐constructed 10 × Genomics barcodes (10 × Genomics (Shanghai) Co., Ltd), individual cells, and a Master Mix. Within these GEMs, cell lysis and reverse transcription occur, during which the 10X Genomics barcodes were ligated to the cDNA products derived from the respective cells. Subsequently, the GEMs were disrupted, facilitating the mixing of cDNAs from different cells. Following this, PCR amplification and quality control procedures were implemented to ensure the integrity of the resultant products. Upon successful completion of quality control, sequencing libraries were generated. Initially, the cDNA was chemically fragmented into pieces ranging from 200 to 300 base pairs, followed by end repair and the addition of A‐tails. The P7 adaptor was then ligated, and a sample index was incorporated through PCR amplification, resulting in the sorting of fragments to produce a cDNA library. The CellRanger analysis pipeline (version 5.0.0) was employed to process the single‐cell RNA sequencing data generated by 10 × Genomics. This process involves aligning the reads to a reference genome and constructing a matrix in which each cell and gene was quantified using unique molecular identifiers, referred to as the gene expression matrix. Normalization of feature counts was performed by dividing the UMI count for each cell by the total count, scaling to a factor of 10000, and applying a natural logarithm transformation. To reduce the dimensionality of the gene expression matrix, PCA was conducted, followed by the use of scree plots to ascertain the optimal number of components. Monocle3 was used to perform cell trajectory analysis.^[^ ^75^ ^]^

### Statistical Analysis

Statistical analysis and graphical representation were conducted utilizing GraphPad Prism (version 8.0) and R (version 4.0.3). An unpaired Student's t‐test was employed to compare the two groups. The results were expressed as mean ± standard deviation. Asterisks denote statistical significance (ns, no significance; *p < 0.05; **p < 0.01; ***p < 0.001; ****p < 0.0001).

## Conflict of Interest

The authors declare no conflict of interest.

## Author Contributions

D.C., L.S., J.C., and Z.Y. contributed equally to this work. J.Y., D.C., X.L., and Y.Z. conceptualized and supervised this study. D.C., J.Y., L.S., J.C., and Z.Y. participated in the overall experiments and wrote the draft. X.Z., H.L., L.W., G.Z., and Q.Z. performed the experiments and downloaded the data. G.J., Y.J., Y.X., H.L., and D.Y. collected and analyzed data. R.C., H.Z., and R.Z. created the figures. J.Y., D.C., L.S., X.L., and Y.Z. verified the data and revised the manuscript. All authors read and approved the final manuscript.

## Supporting information



Supporting Information

## Data Availability

The data that support the findings of this study are available from the corresponding author upon reasonable request. The mass spectrometry proteomics data have been deposited to the iProX repository (a member of the ProteomeXchange consortium) under the dataset identifier PXD070137. The CyTOF data are available in the NGDC OMIX database under accession code OMIX012640.
