# Supplementary material for: Single‐Cell Transcriptome Atlas Reveals the Underlying Mechanism of Kynurenic Acid in the Regulation of Tumor Immune Microenvironment in Glioblastoma
Source: Adv Sci (Weinh). 2025 Nov 12;13(5):e07705. doi: 10.1002/advs.202507705 (PMC12849964; doi:10.1002/advs.202507705)
Supplement: Supplementary file 1 — Supporting Information [file ADVS-13-e07705-s001.docx]

***Supplementary File***

**Single-cell transcriptome atlas reveals the underlying mechanism of kynurenic acid in the regulation of tumor immune microenvironment in glioblastoma**

Di Chen *et al.*

**The supplementary file includes:**

**Supplementary Figure 1-8**


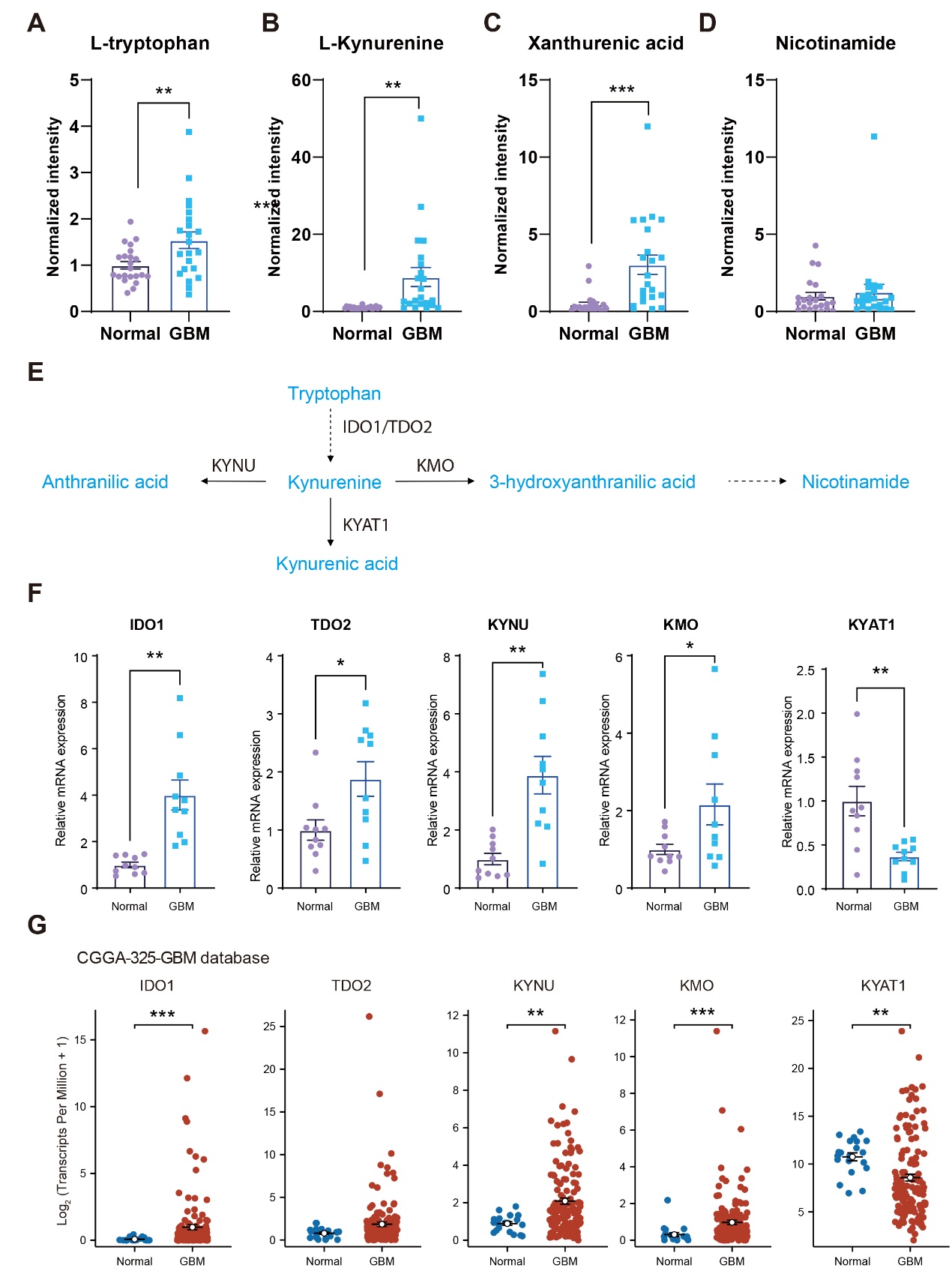


**Fig. S1. Dysregulation of tryptophan metabolism and** **enzyme expression in GBM.**

(A-D) Levels of L-tryptophan (A), L-kynurenine (B), xanthurenic acid (C), and nicotinamide (D) in GBM and normal brain tissues (Normal = 22, GBM = 22).
(E) Schematic of the kynurenine metabolic pathway.

(F) Relative mRNA expression levels of IDO1, TDO2, KMO, KYNU, and KYAT1 in GBM and normal brain tissues (Normal = 10, GBM = 10).

(G) Validation of IDO1, TDO2, KMO, KYNU, and KYAT1 expression in the CGGA-325 GBM cohort.


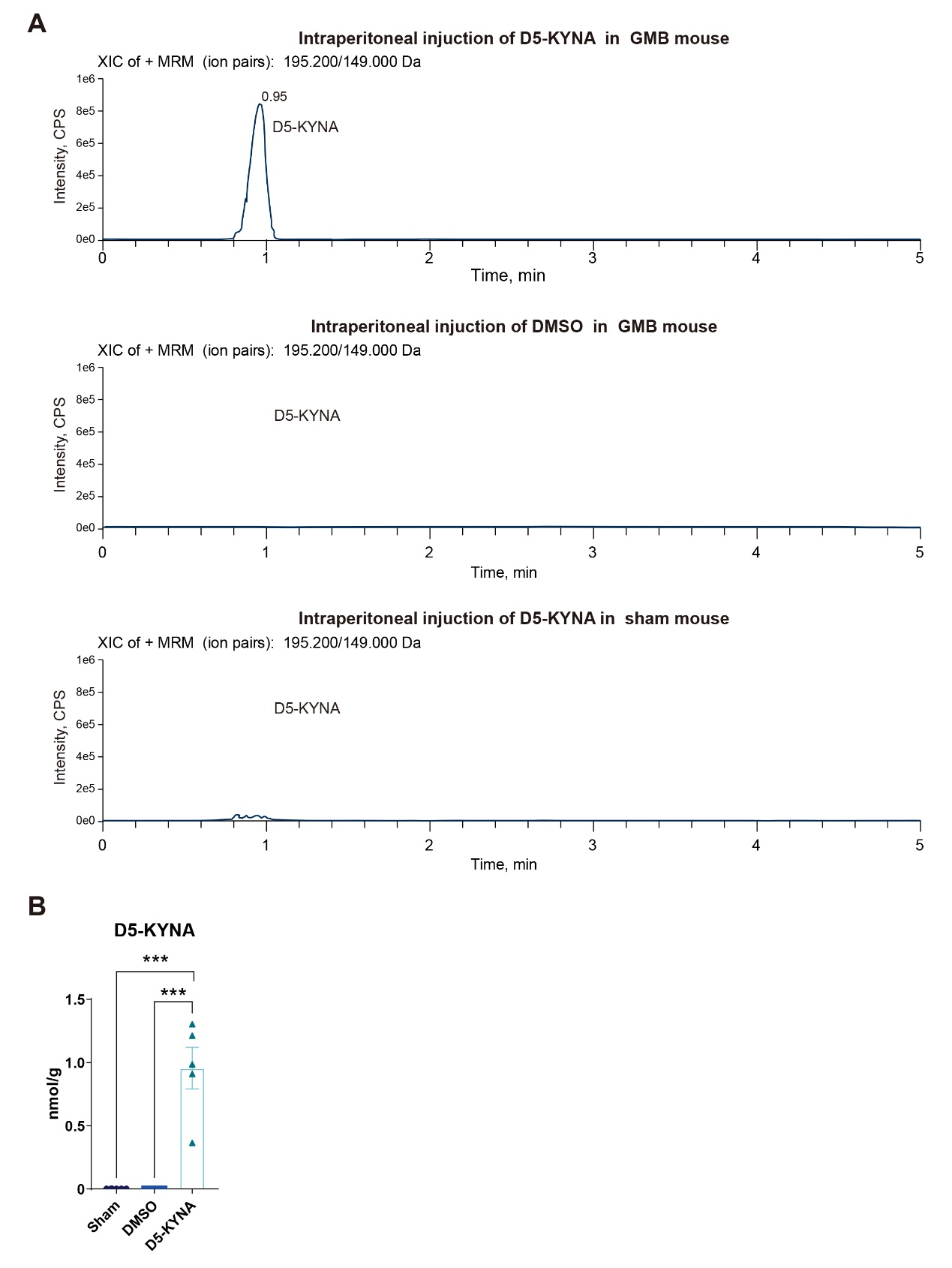


**Fig. S2. Targeted mass spectrometry detection of intracranial D5-KYNA levels in GBM-bearing mice following systemic administration.**

(A) Extracted ion chromatograms of targeted mass spectrometry showing D5-KYNA levels in GBM tissues at 1-minute post-injection (n = 5).

(B) Quantitative analysis of D5-KYNA levels in GBM tissues (n = 5).


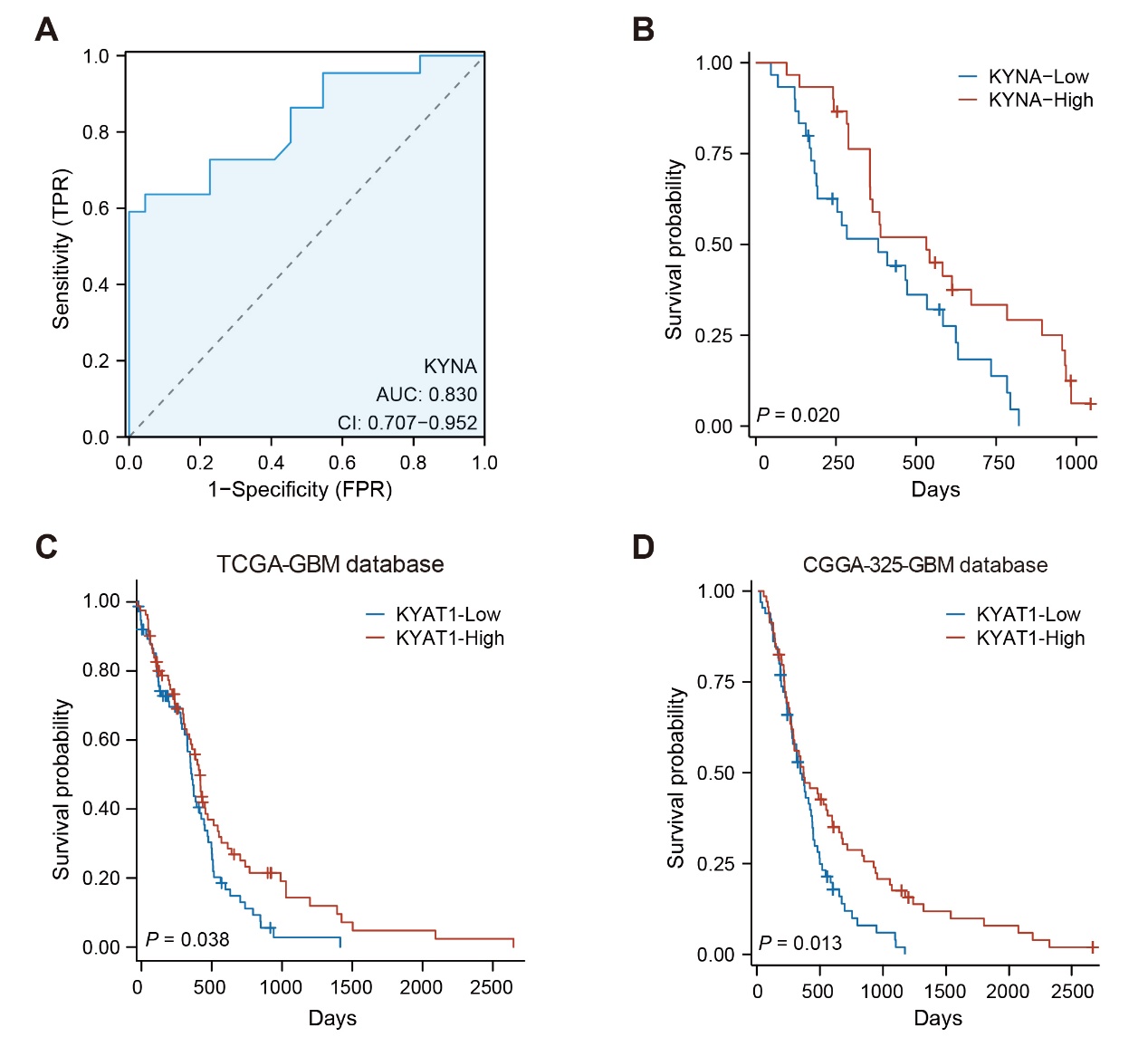


**Fig. S3. Diagnostic and prognostic value of KYNA in GBM.**

(A) In cohort 1, receiver operating characteristic (ROC) curve analysis of KYNA for diagnosing GBM, with an area under the curve (AUC) at 0.830 (Normal = 22, GBM = 22).

(B) In cohort 2, Kaplan-Meier survival analysis showing the correlation of higher KYNA levels with prolonged overall survival (n = 60).

(C-D) Kaplan-Meier survival analysis of the TCGA-GBM (C) and CGGA-325-GBM (D) databases showing the correlation of higher KYAT1 levels with prolonged overall survival.


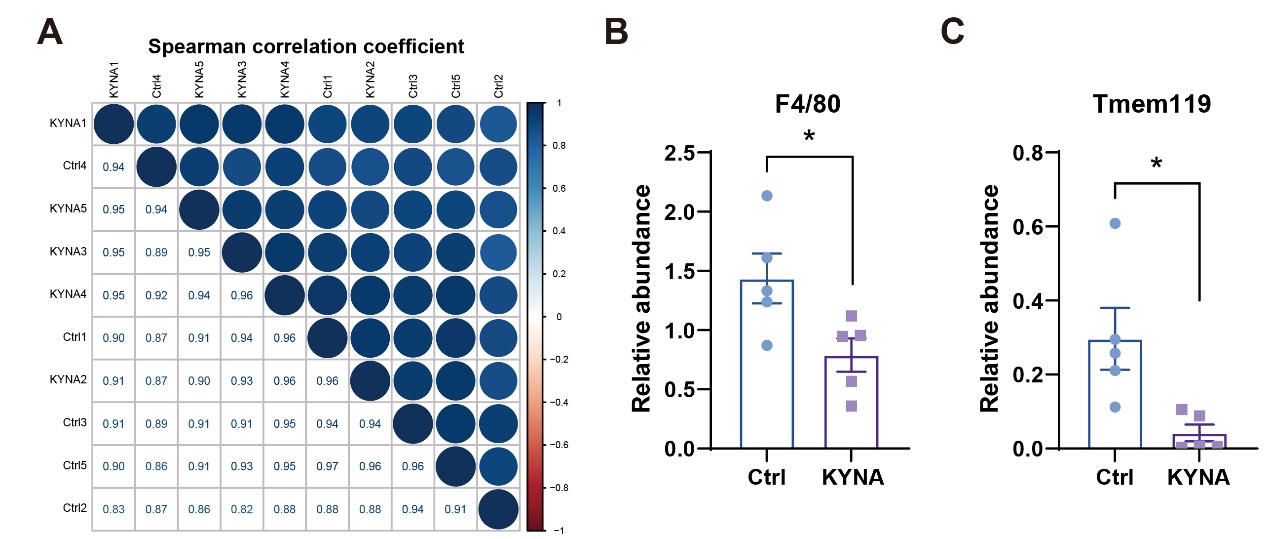


**Fig. S4. Proteomics revealed dysregulated tryptophan metabolism and immune alterations in GBM.**

(A) Spearman correlation coefficient analysis of proteomic data (n = 5).
(B-C) Bar plots depicting the relative abundance of F4/80 (B) and Tmem119 (C) in Vehicle- and KYNA-treated GBM tissues (n = 5).


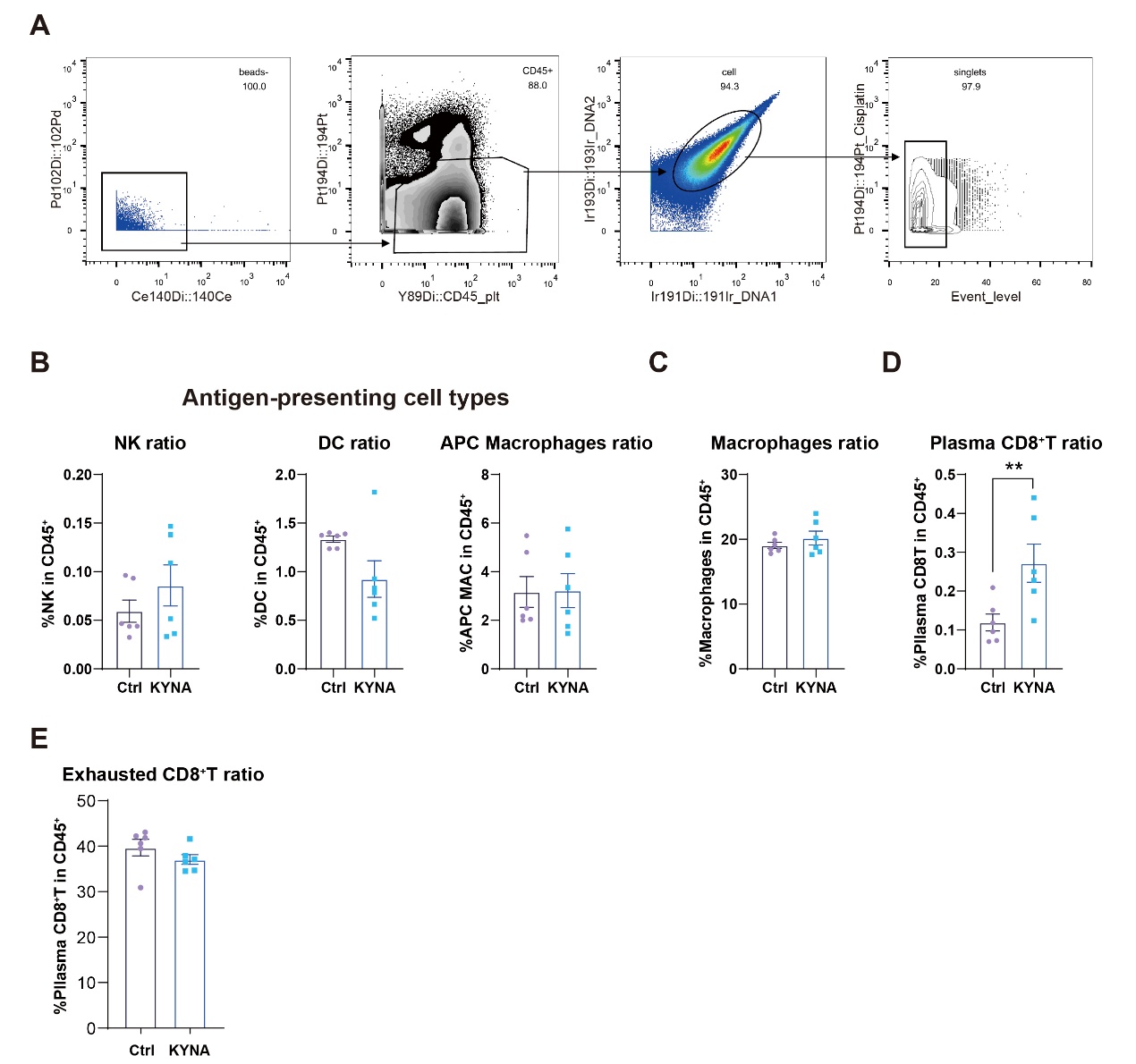


**Fig. S5. Gating strategy and CyTOF analysis of myeloid and adaptive immune cells.**(A) Gating strategy for CyTOF analysis (n = 6).
(B-E) Percentage change of antigen-presenting cells (B), macrophages (C), plasma CD8^+^ T cells (D) and exhausted CD8^+^ T cells (E) in CD45^+^ cells from CyTOF data (n = 6).


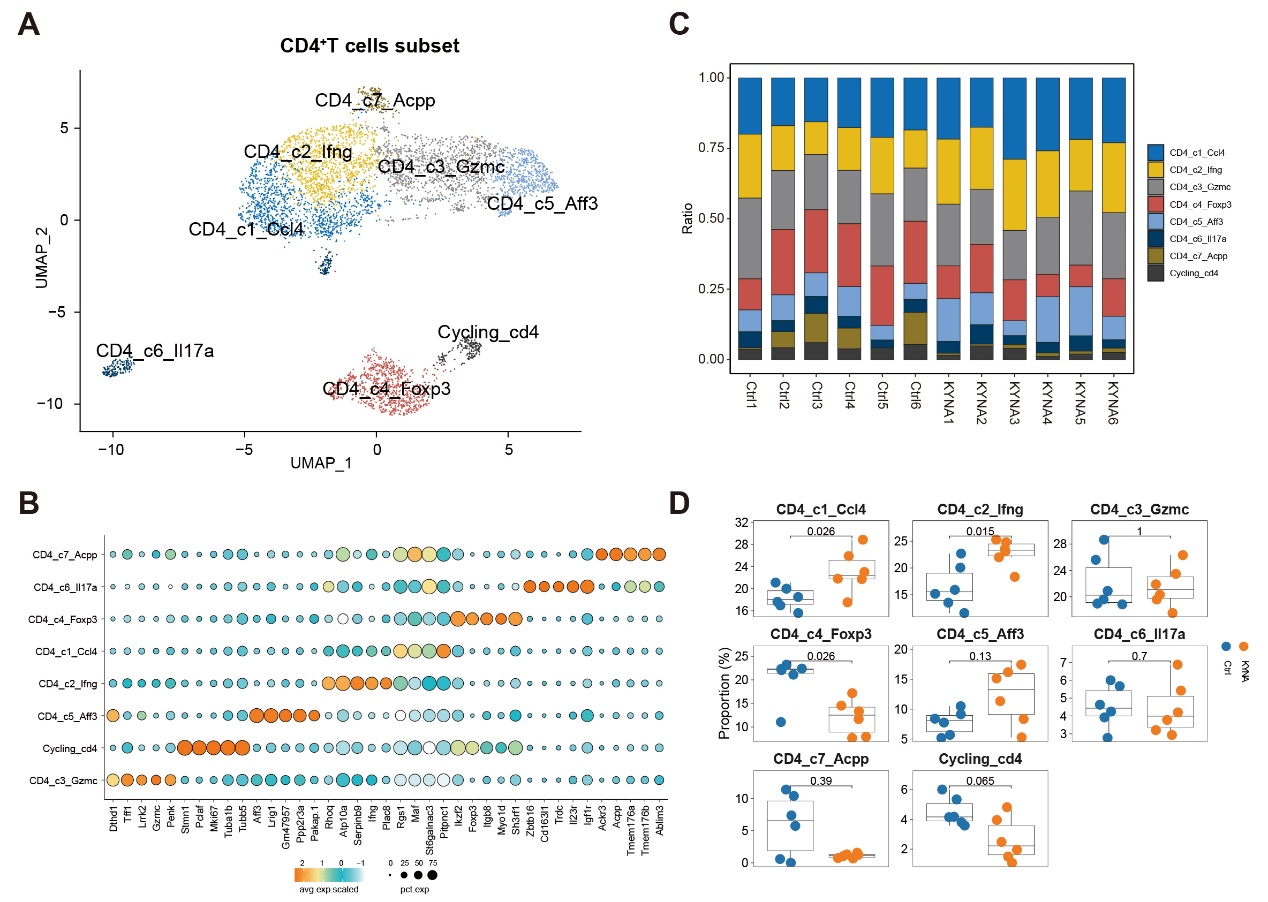


**Fig. S6. scRNA-seq analysis of CD4^+^ T cell lineages after KYNA treatment.**(A) UMAP visualization of 8 CD4^+^ T cell clusters.
(B) Dot plot showing the expression of characteristic marker genes in the CD4^+^ T cell clusters.
(C) Stacked bar plot depicting the proportions of CD4^+^ T cell clusters (n = 6).
(D) Box plots quantifying the differences in the proportions of CD4^+^ T cell clusters (n = 6).


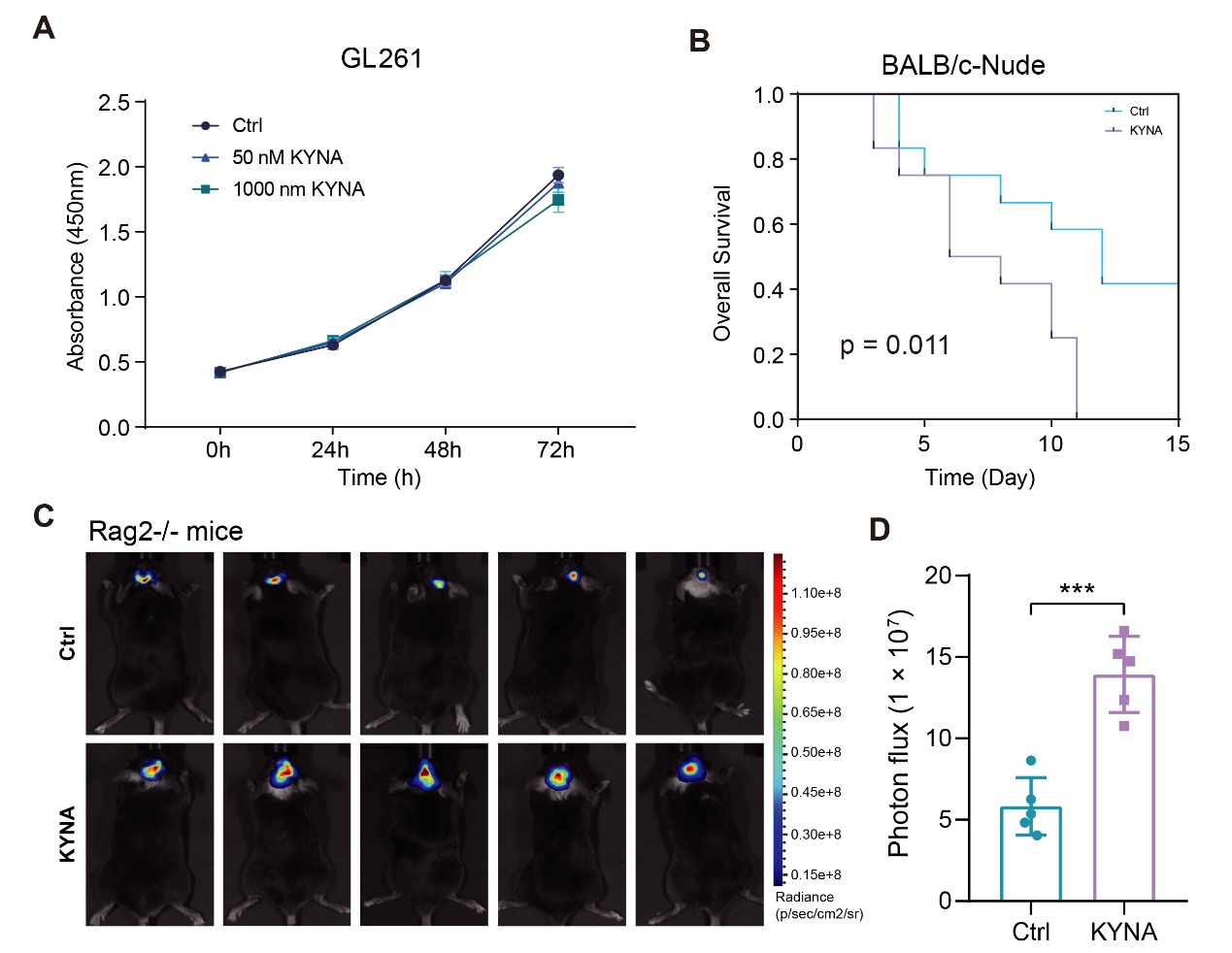


**Fig. S7.** **KYNA exerts anti-tumor effects dependent on adaptive immune cells in GBM models.**

(A) CCK8 proliferation assay of GL261 GBM cells treated with Vehicle (Ctrl), 50 nM KYNA, or 1000 nM KYNA (n = 8).

(B) Kaplan-Meier survival analysis of BALBc-Nude mice with GL261 GBM (n = 9).

(C) Bioluminescent imaging of GBM-bearing Rag2-/- mice treated with KYNA or Vehicle (n = 5).
(D) Quantification of photon flux from bioluminescent imaging shown in (C) (n = 5).

.


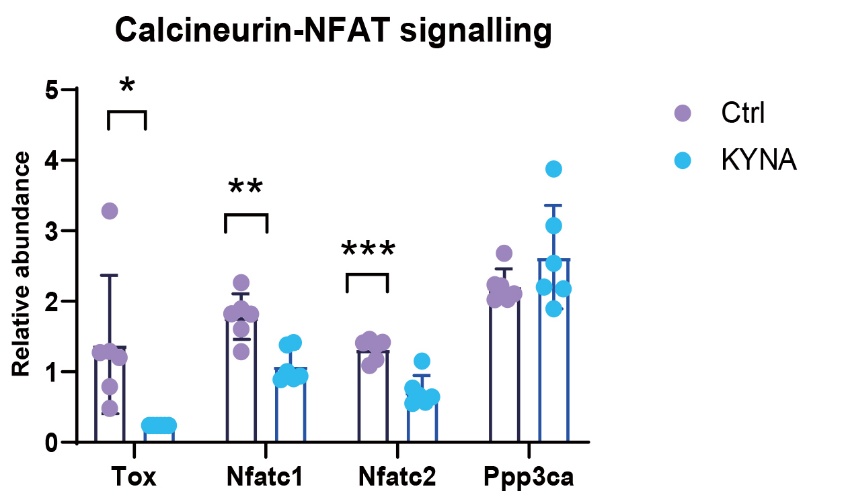


**Fig. S8. Relative abundance of key genes within the Calcineurin-NFAT signaling pathway in Ctrl and KYNA groups (n = 6).**
